# Supplementary material for: 'Palaeoshellomics’ reveals the use of freshwater mother-of-pearl in prehistory
Source: eLife. 2019 May 7;8:e45644. doi: 10.7554/eLife.45644 (PMC6542584; doi:10.7554/eLife.45644)

1. Hic74 [*Unio crassus*]

1.1 Position (AA): **172**; Mutation **E** → **Q**

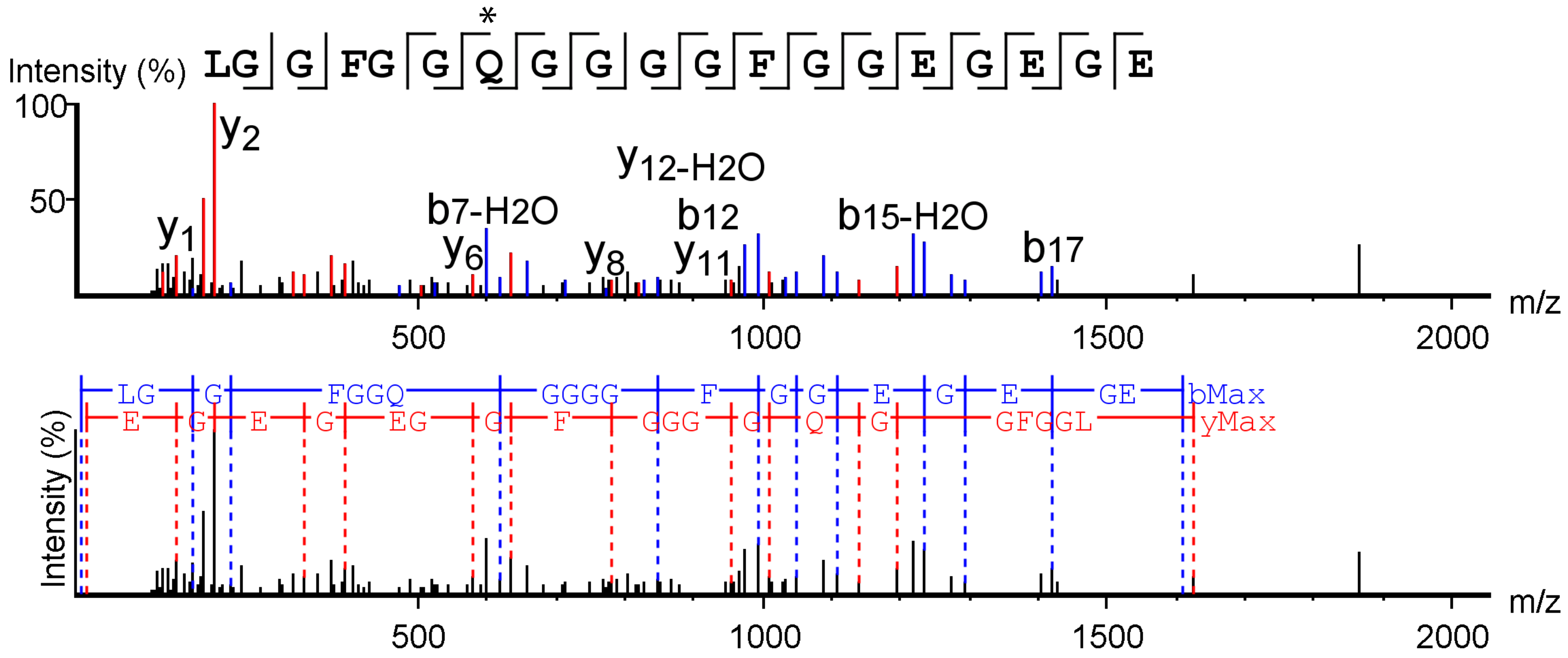

1.2 Position (AA): **310**; Mutation **I** → **F**

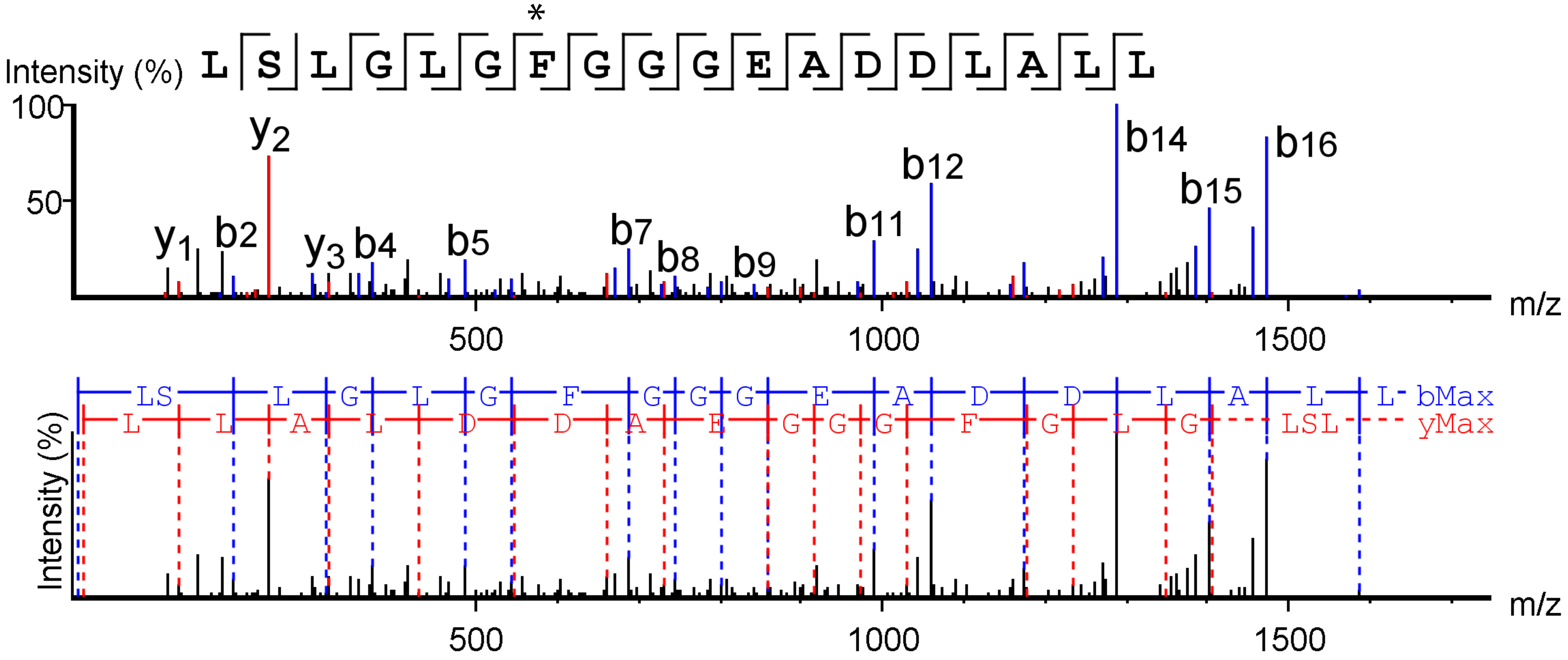

1.3 Position (AA): **310**; Mutation **I** → **F**

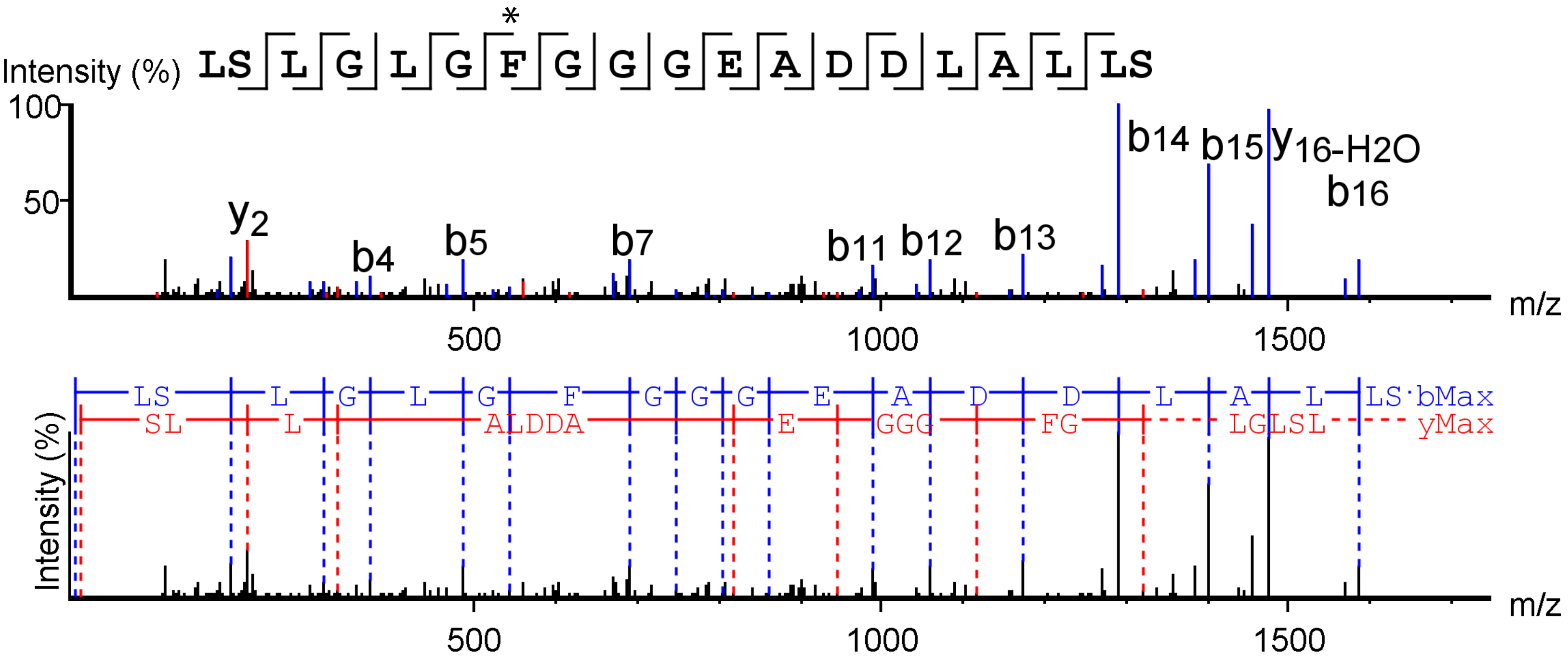

2. Hic74 [*Unio pictorum*]

2.1 Position (AA): **172**; Mutation **E** → **Q**

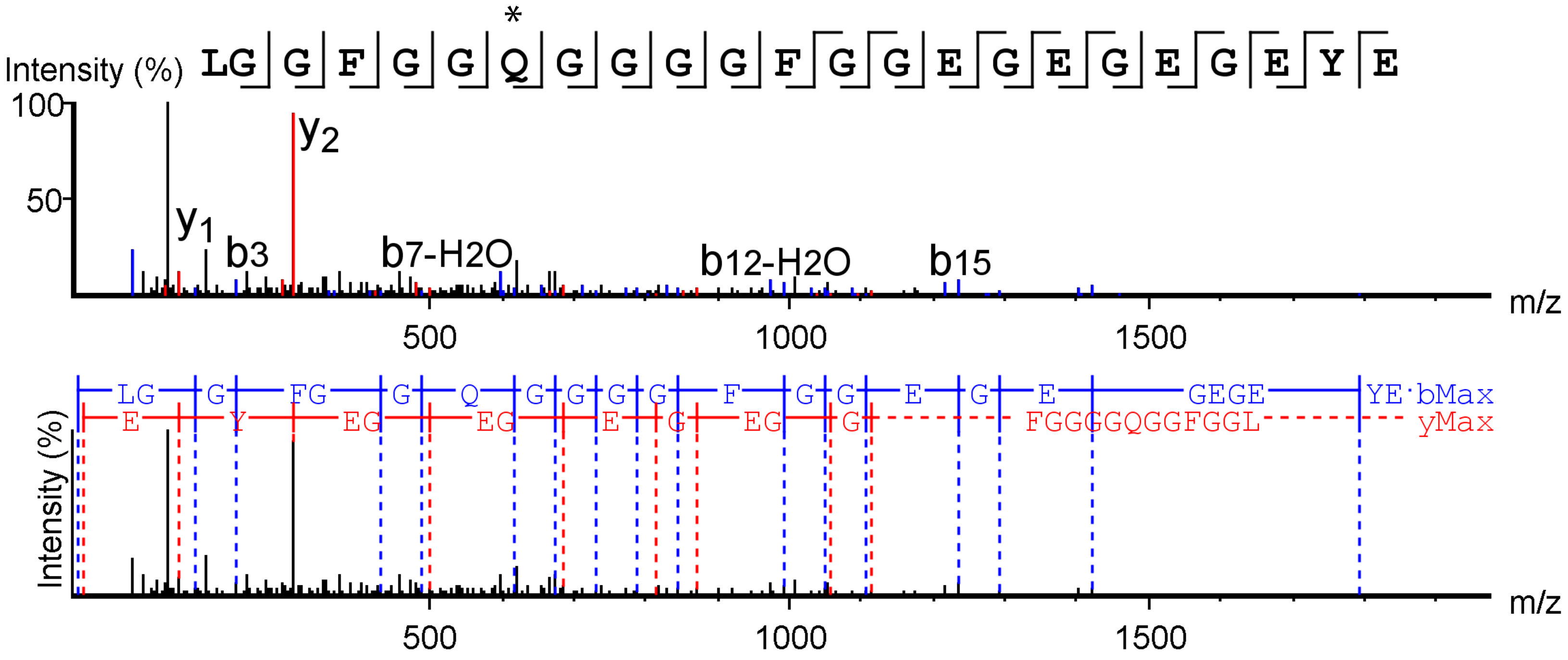

2.2 Position (AA): **310**; Mutation **I** → **F**

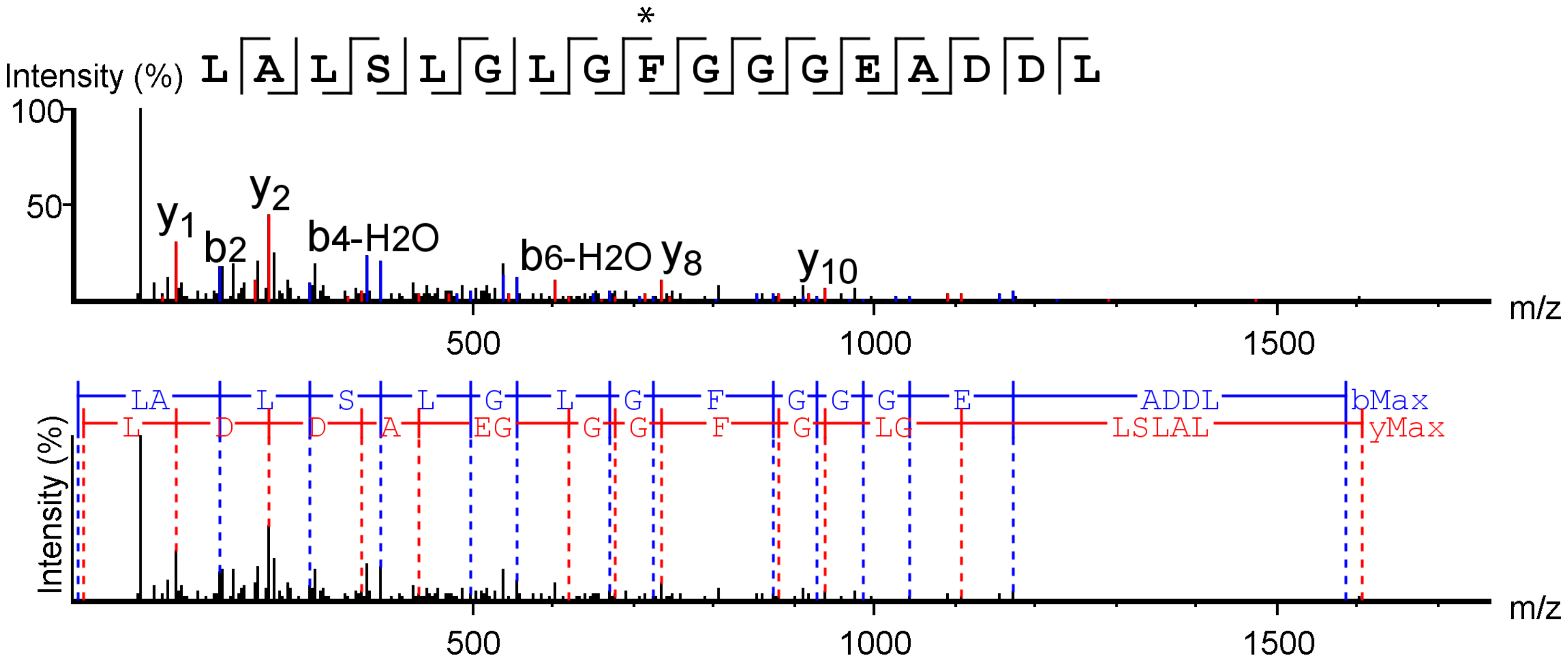

2.3 Position (AA): **310**; Mutation **I** → **F**

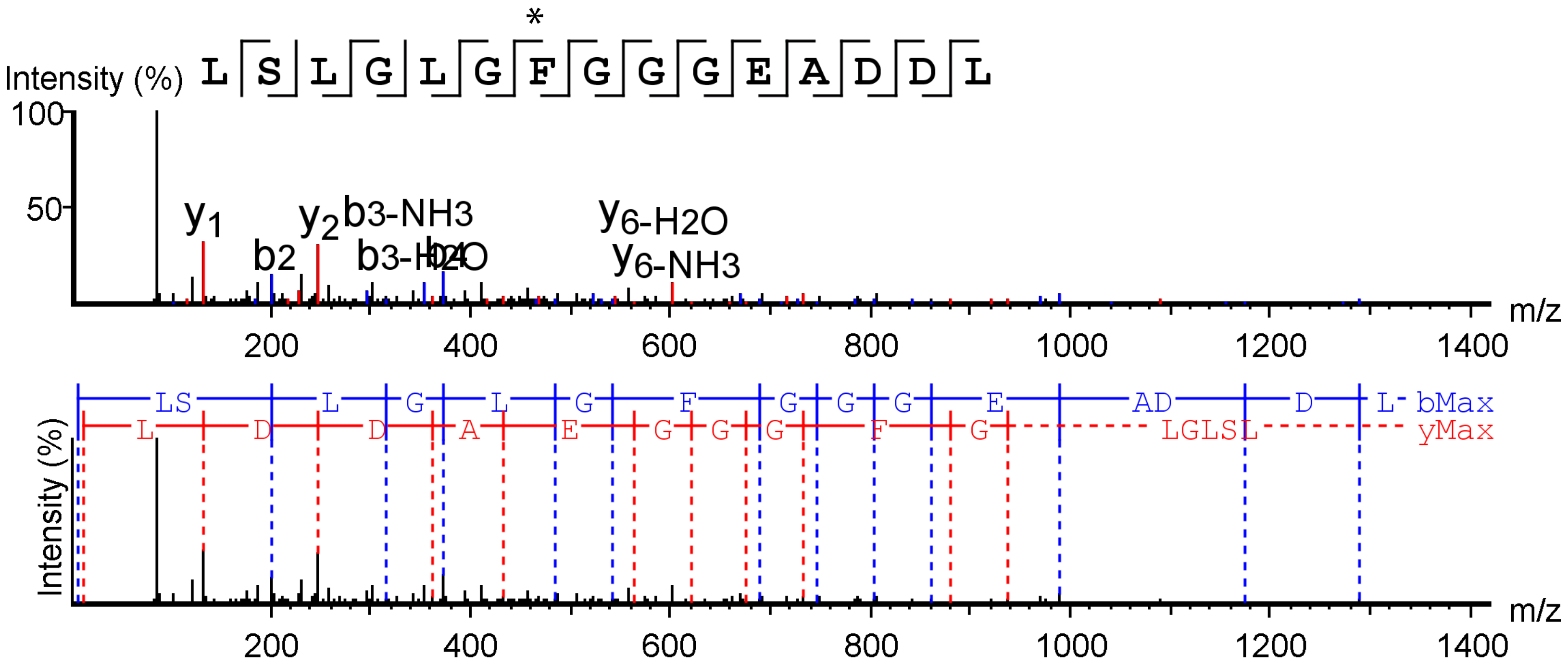

2.4 Position (AA): **801**; Mutation **G** → **S**

804; Mutation **A** → **S**

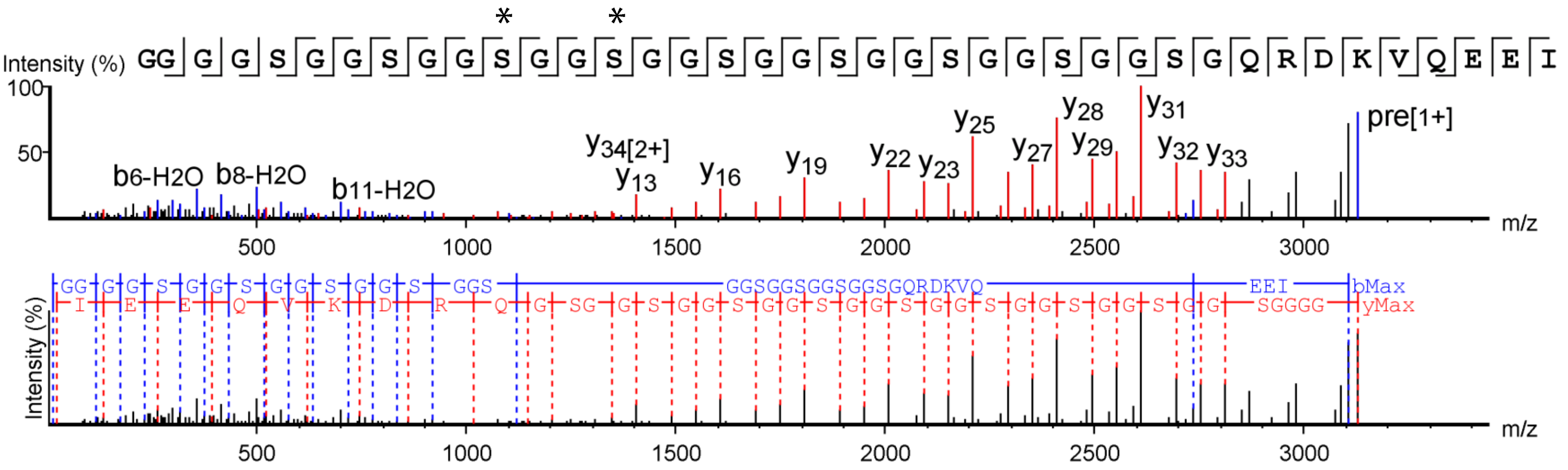

2.5 Position (AA): **801**; Mutation **G → S**

804; Mutation **A → S**

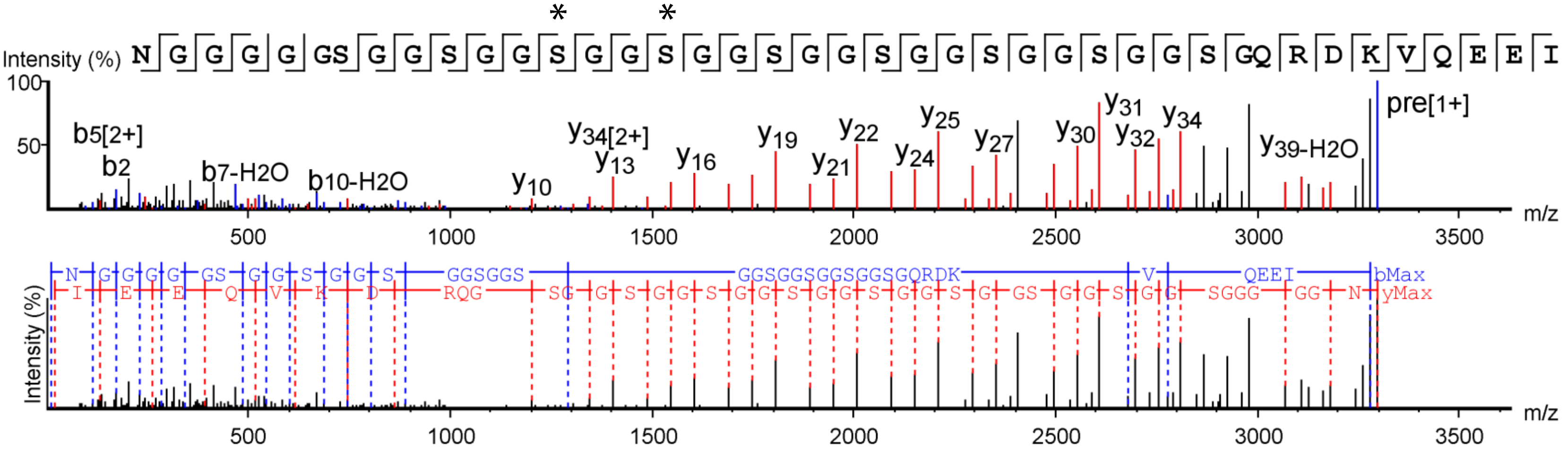

3. Hic74 [*Margaritifera margaritifera*]

3.1 Position (AA): **172**; Mutation **E** → **Q**

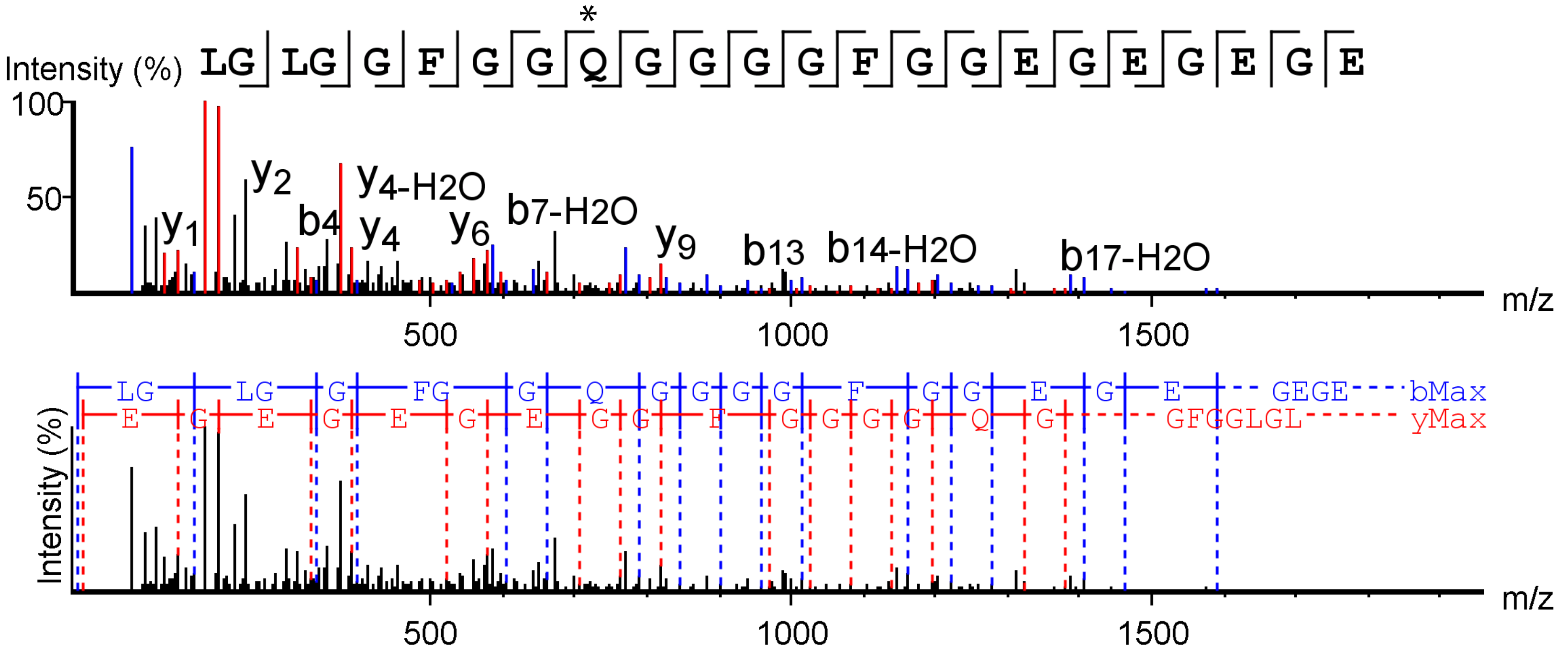

3.2 Position (AA): **172**; Mutation **E** → **Q**

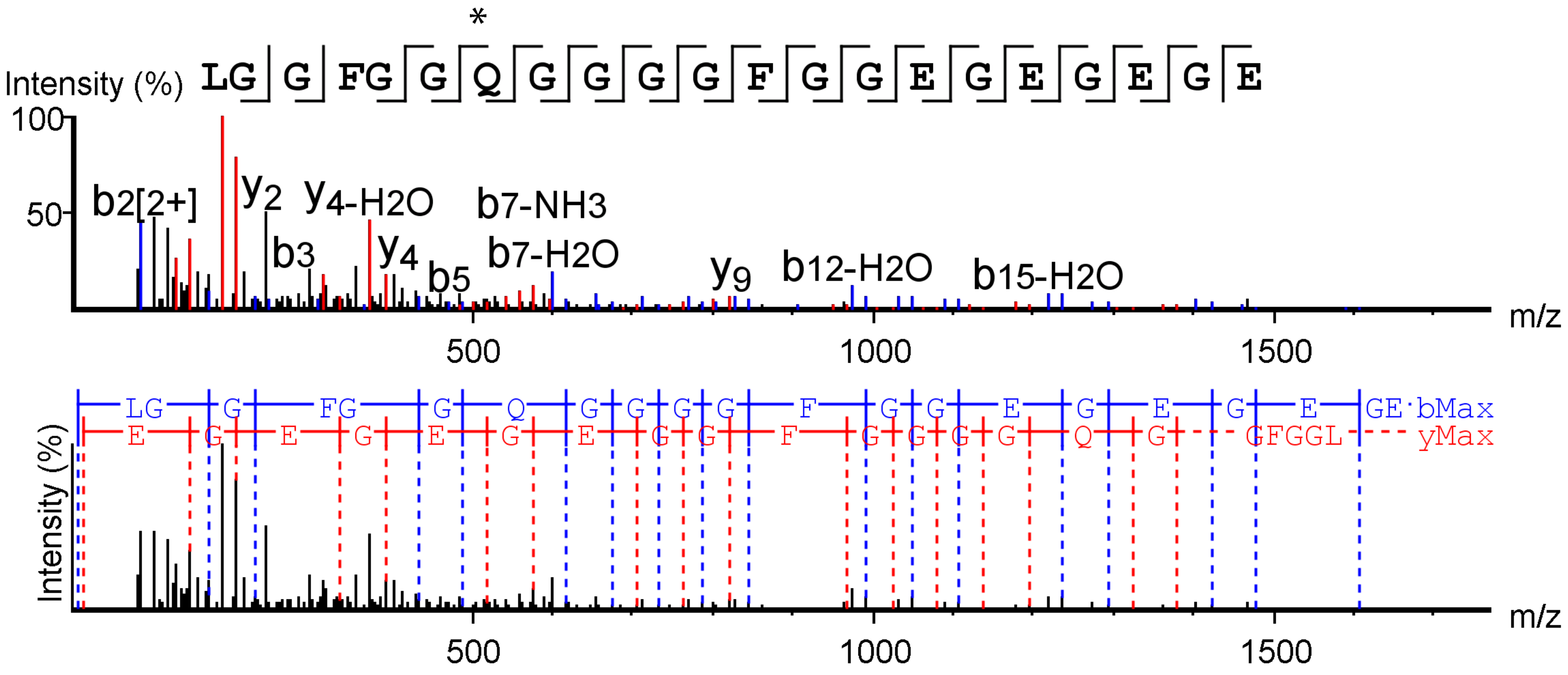

3.3 Position (AA): **289**; Mutation **V** → **L**

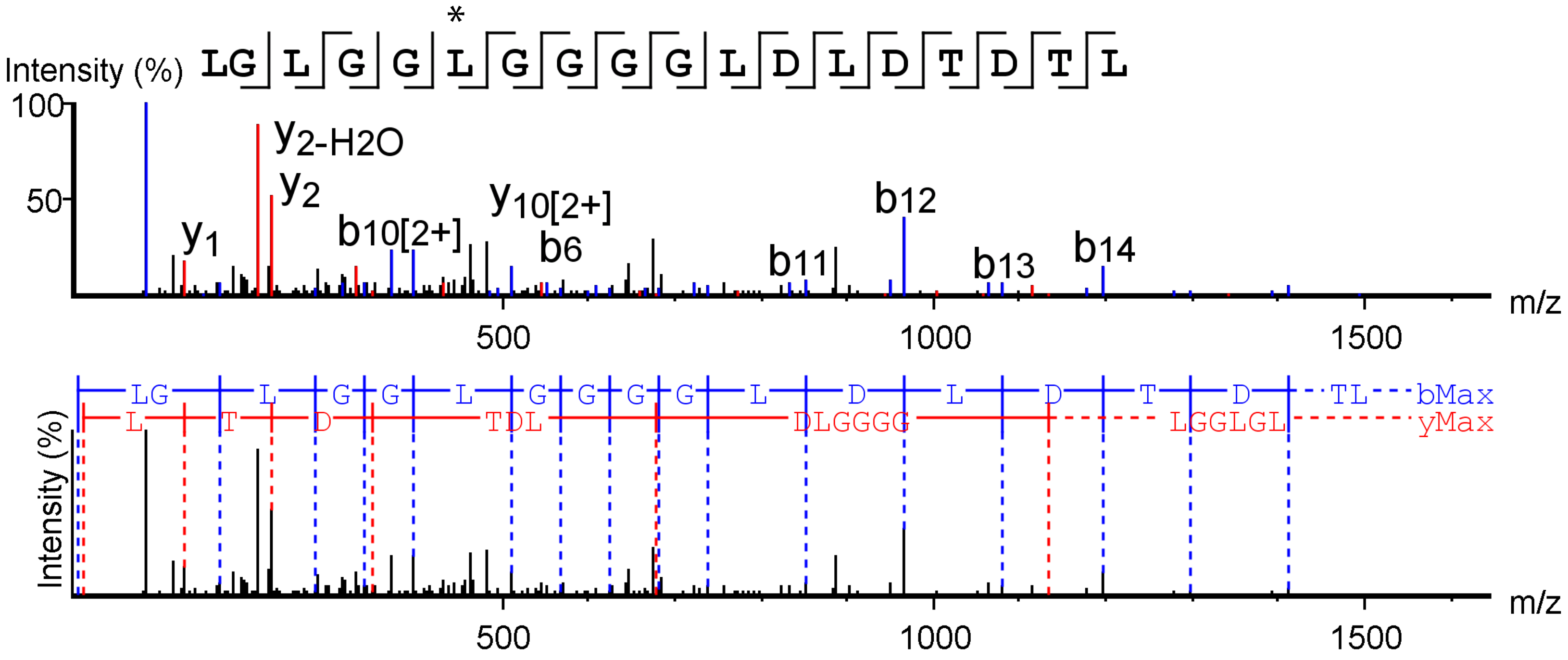

3.4 Position (AA): **289**; Mutation **V** → **L**

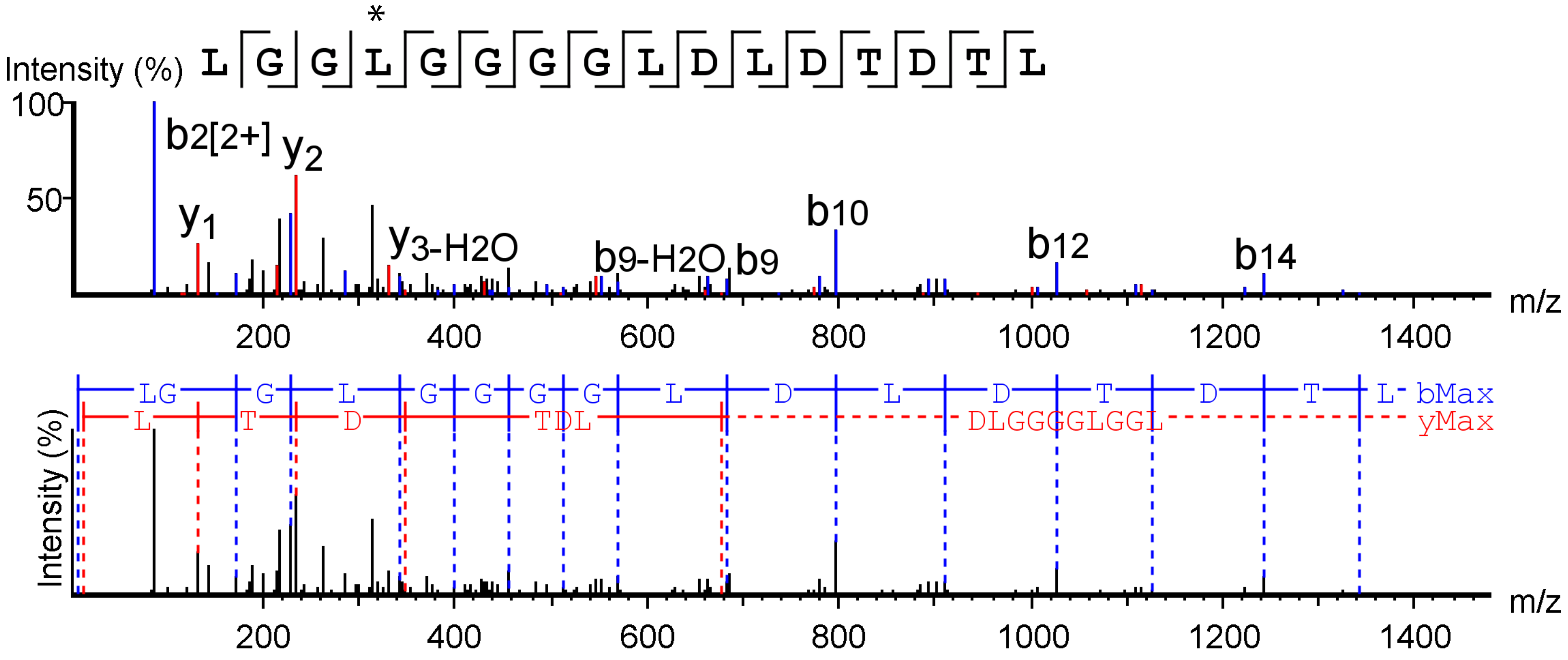

3.5 Position (AA): **310**; Mutation **I** → **F**

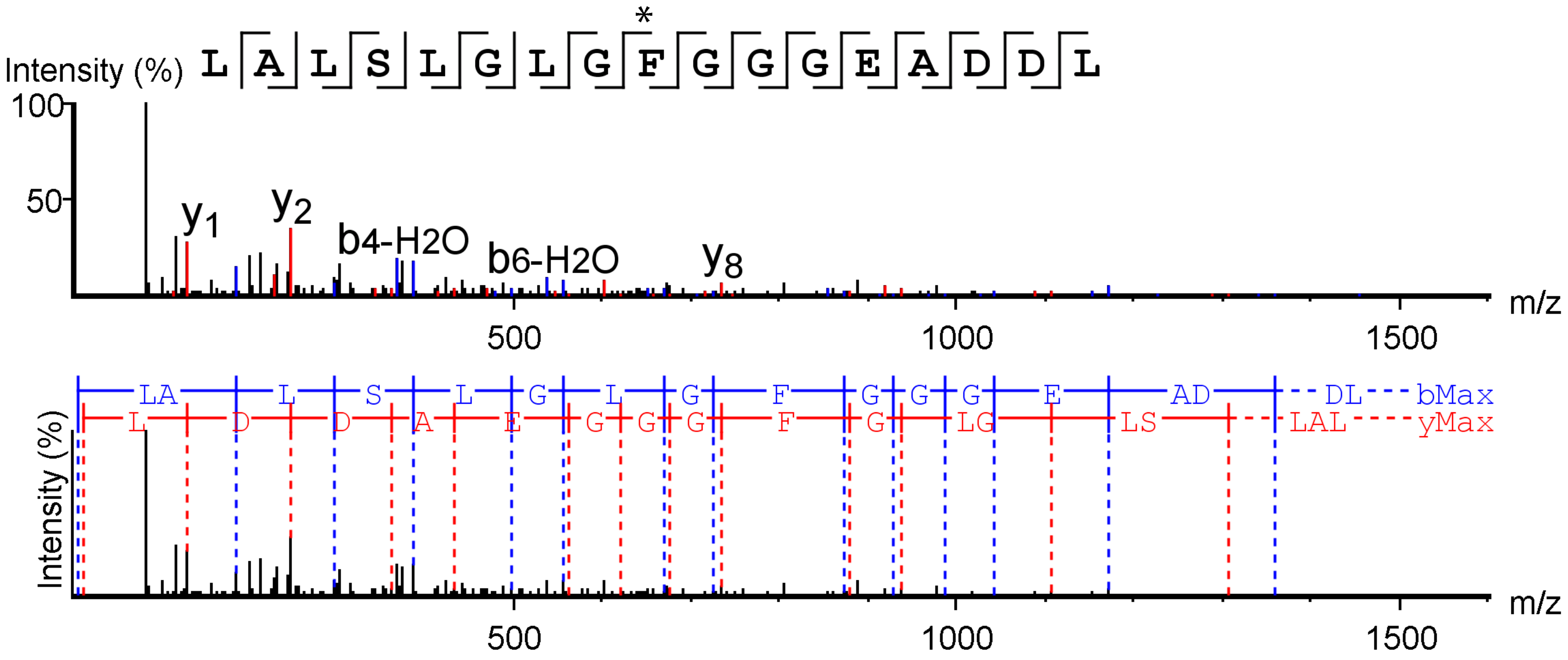

3.6 Position (AA): **310**; Mutation **I** → **F**

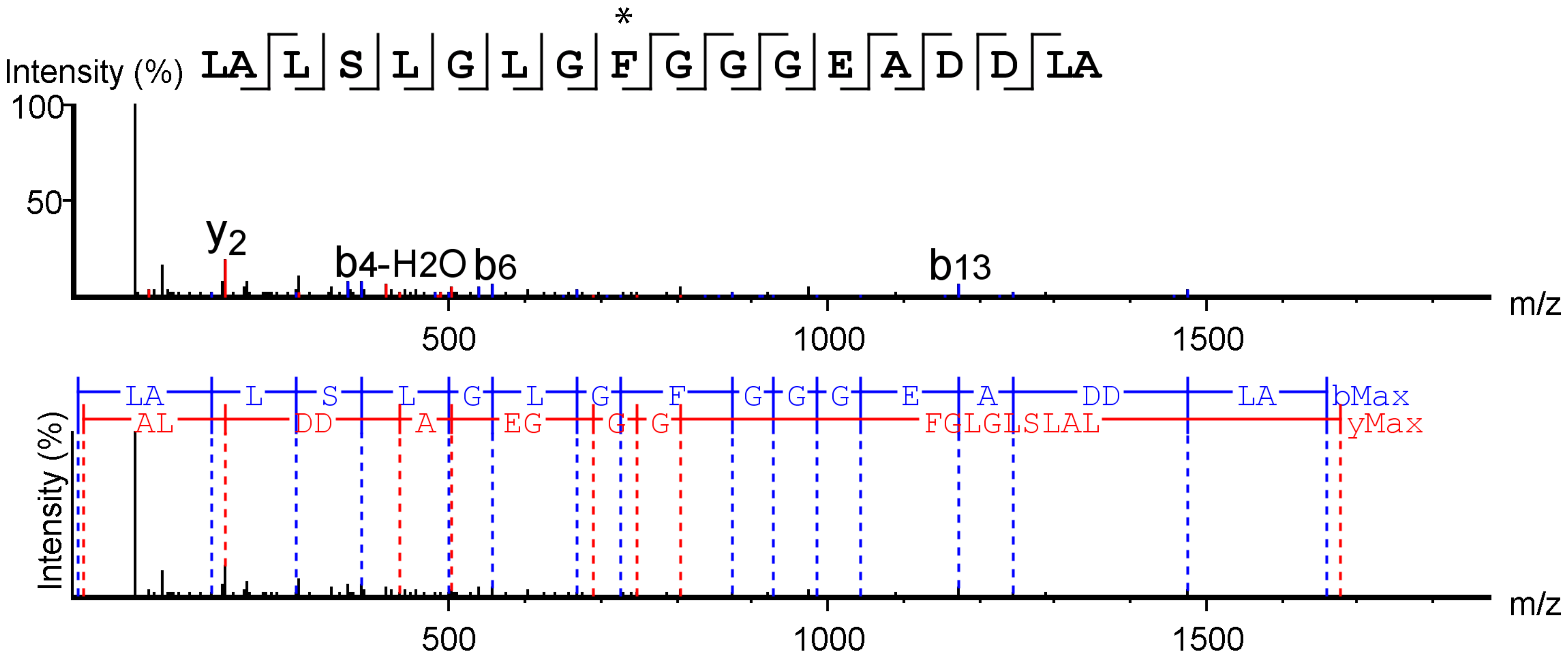

4. Hic74 [*Pseudunio auricularius*]

4.1 Position (AA): **163**; Mutation **S** → **D**

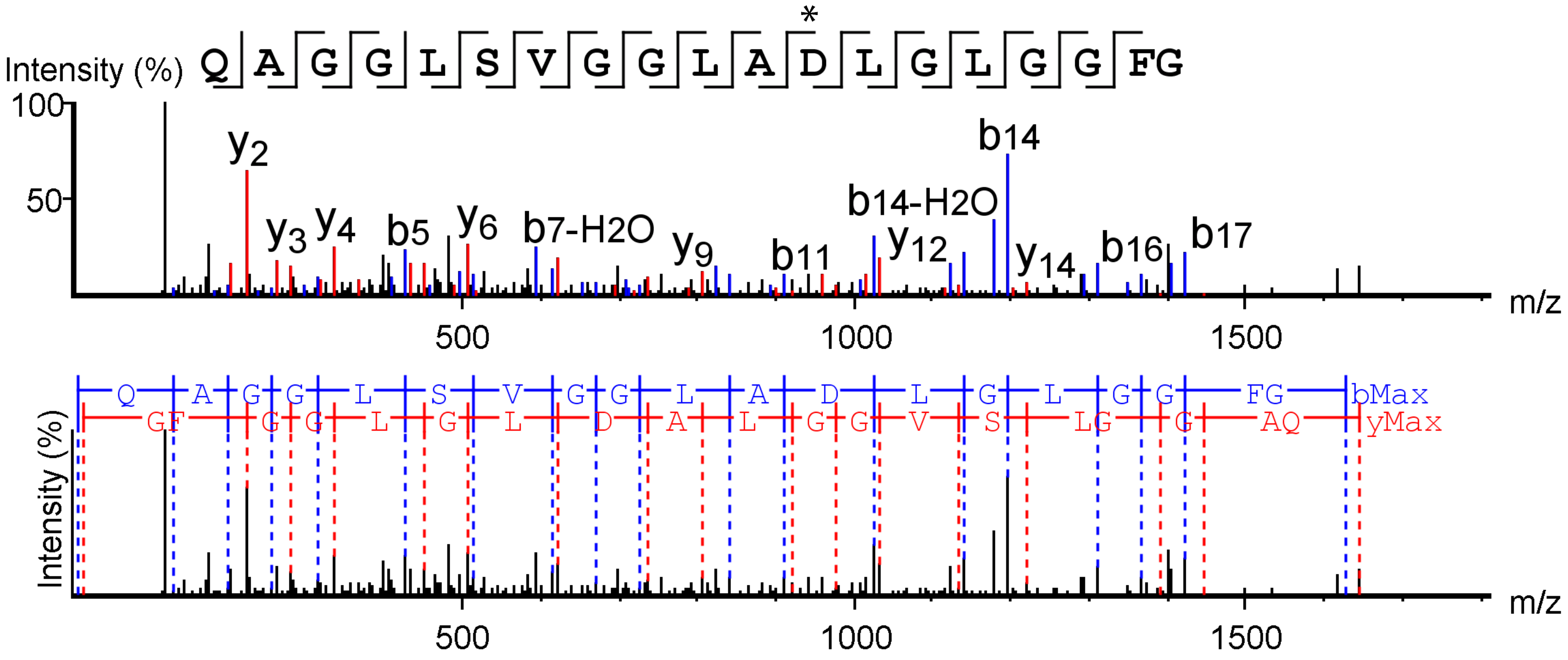

4.2 Position (AA): **163**; Mutation **S** → **D**
175; Mutation **G** → **L**

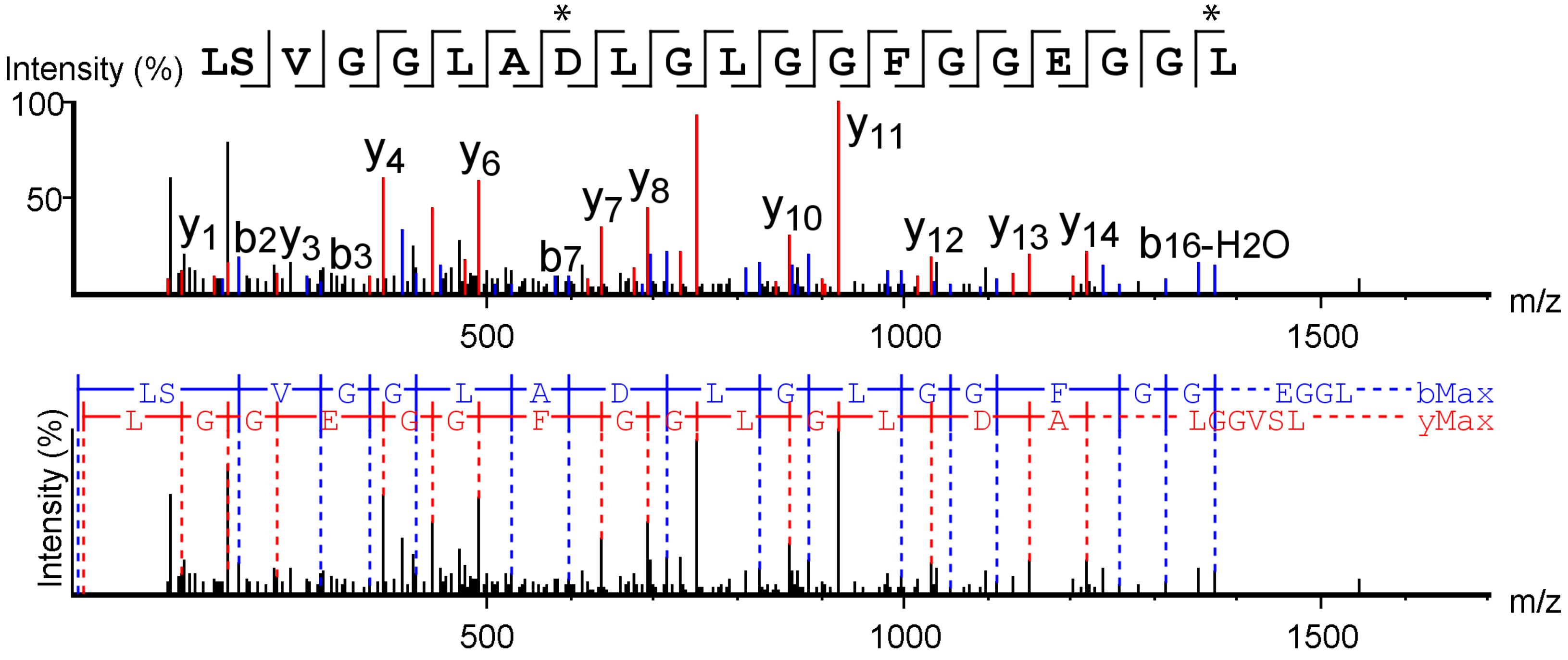

4.3 Position (AA): **163**; Mutation **S** → **D**
175; Mutation **G** → **L**

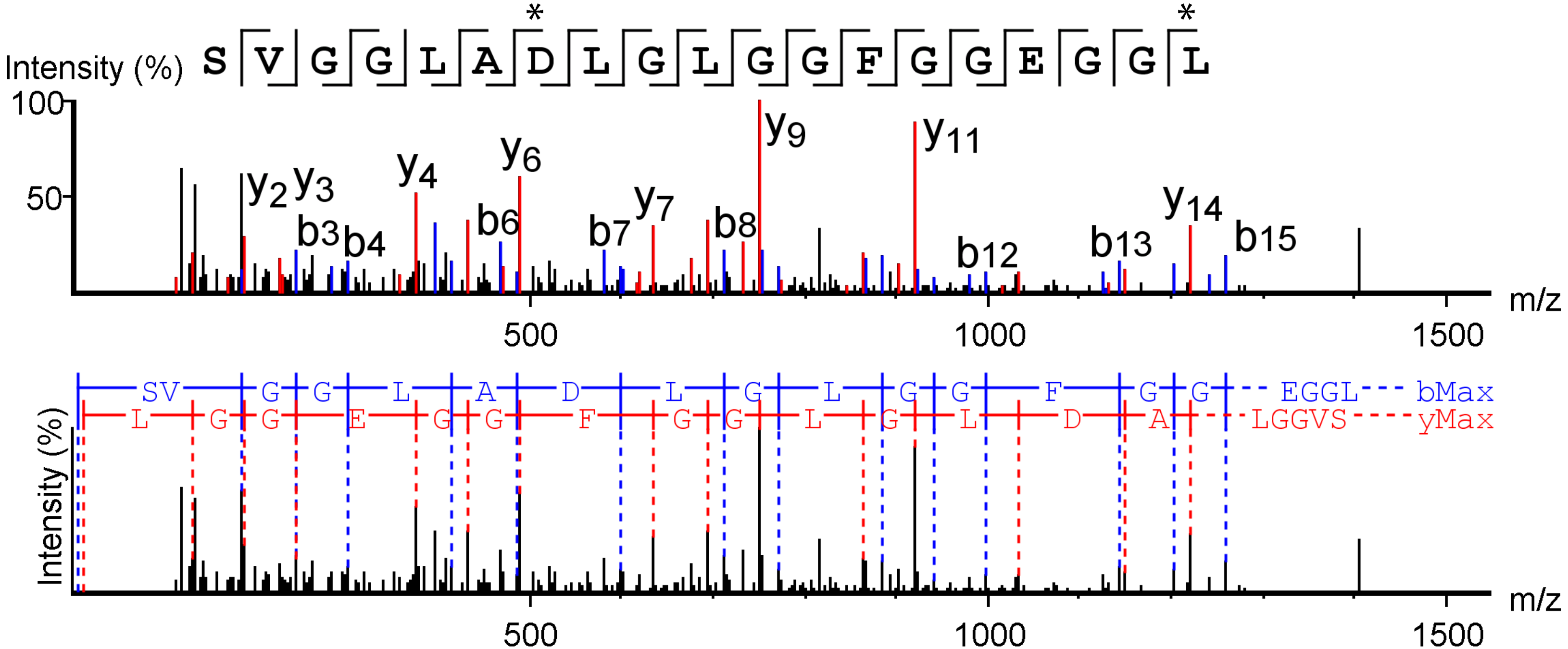

4.4 Position (AA): **284**; Mutation **L** → **F**

292; Mutation **G** → **E**

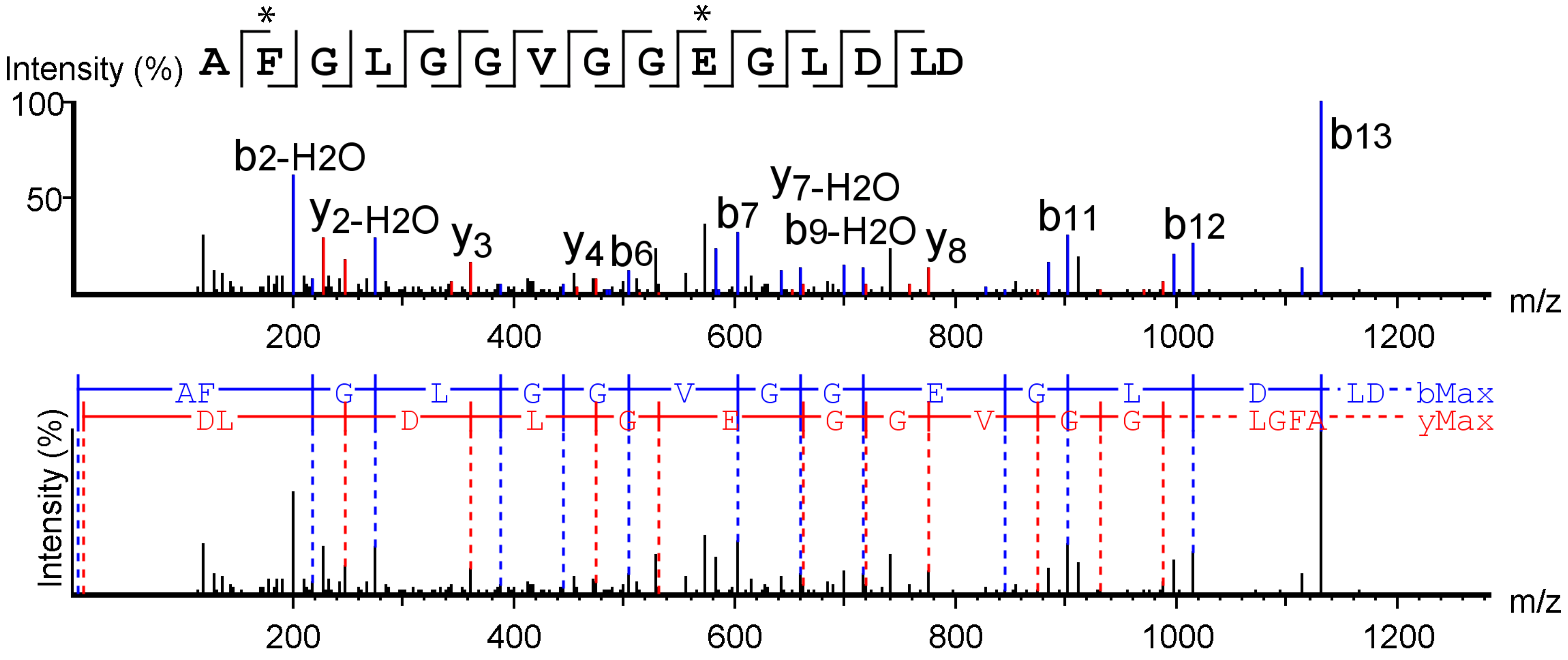

4.5 Position (AA): **284**; Mutation **L** → **F**
292; Mutation **G** → **E**

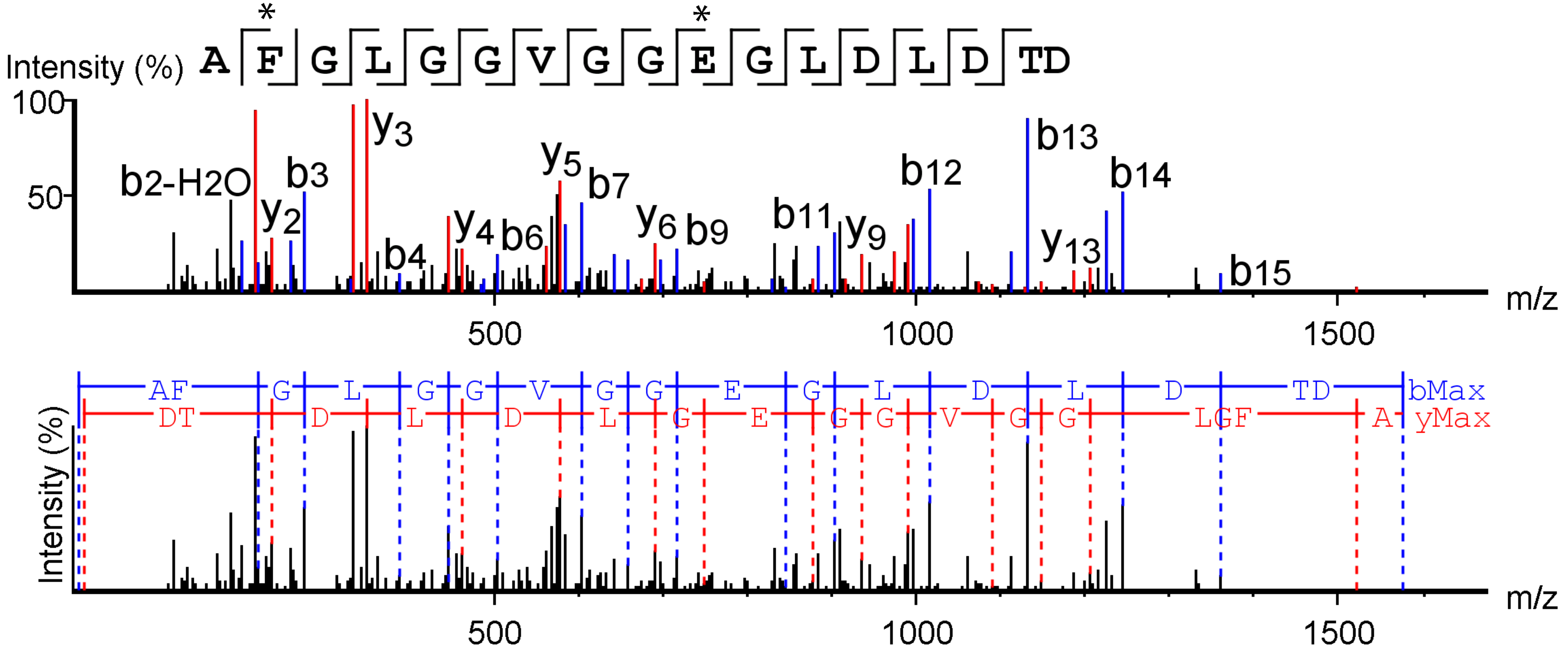

4.6 Position (AA): **306**; Mutation **L** → **F**

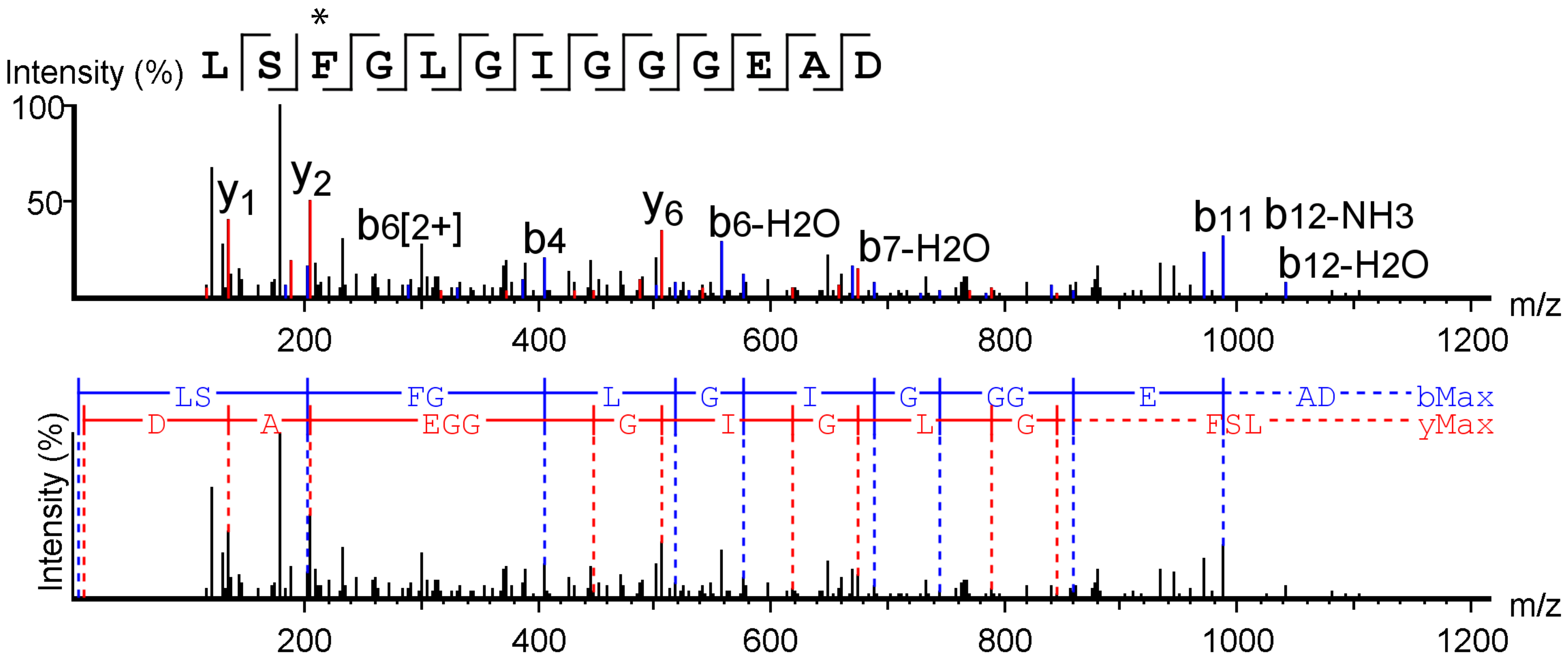

4.7 Position (AA): **306**; Mutation **L** → **F**

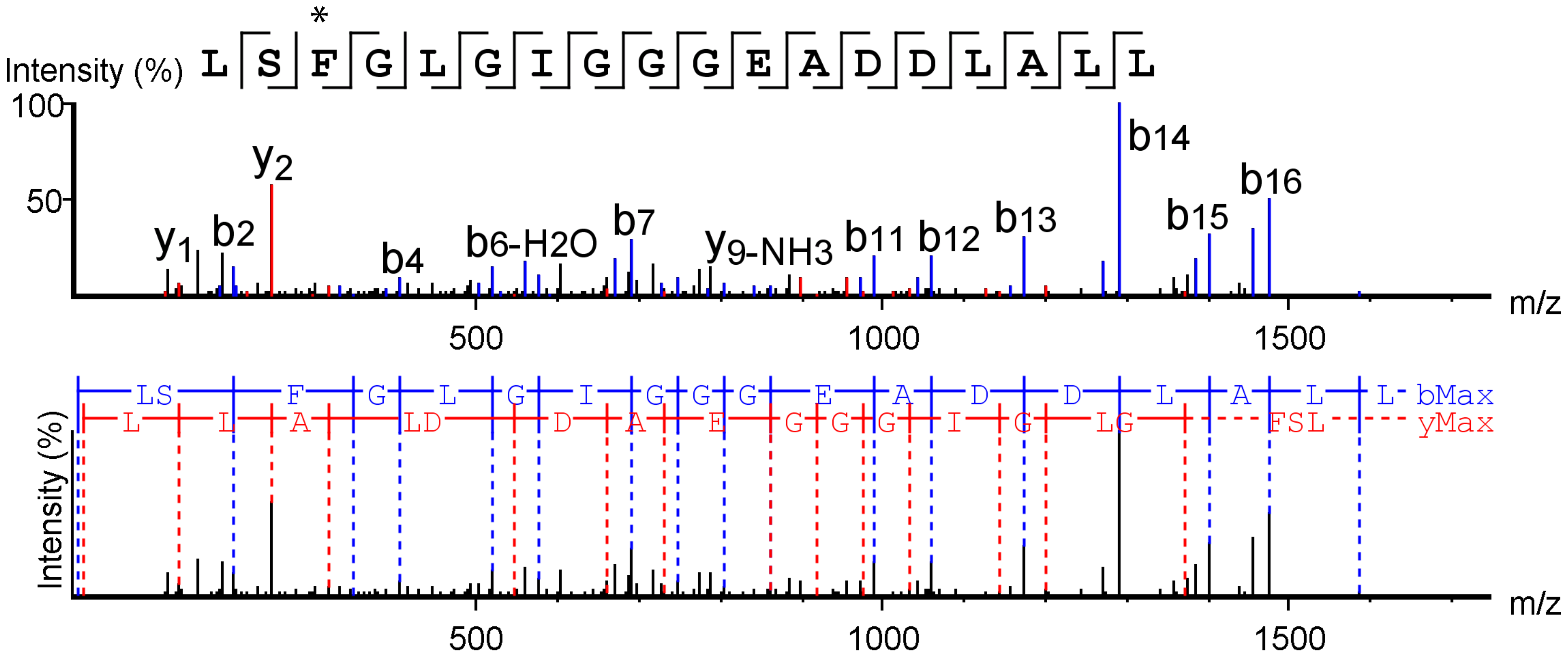

4.8 Position (AA): **821**; Mutation **Q** → **H**
822; Mutation **R** → **H**
827; Mutation **E** → **D**

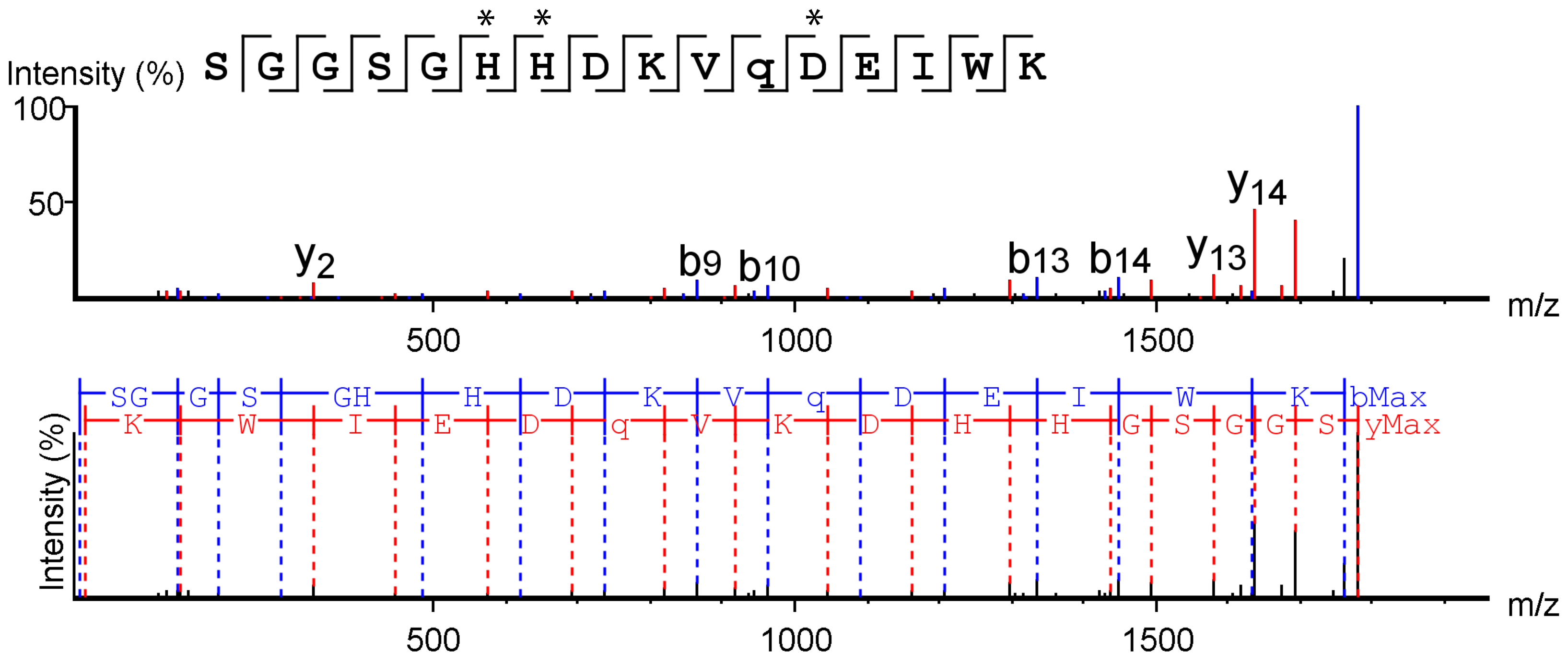

4.9 Position (AA): **821**; Mutation **Q** → **H**
822; Mutation **R** → **H**
827; Mutation **E** → **D**

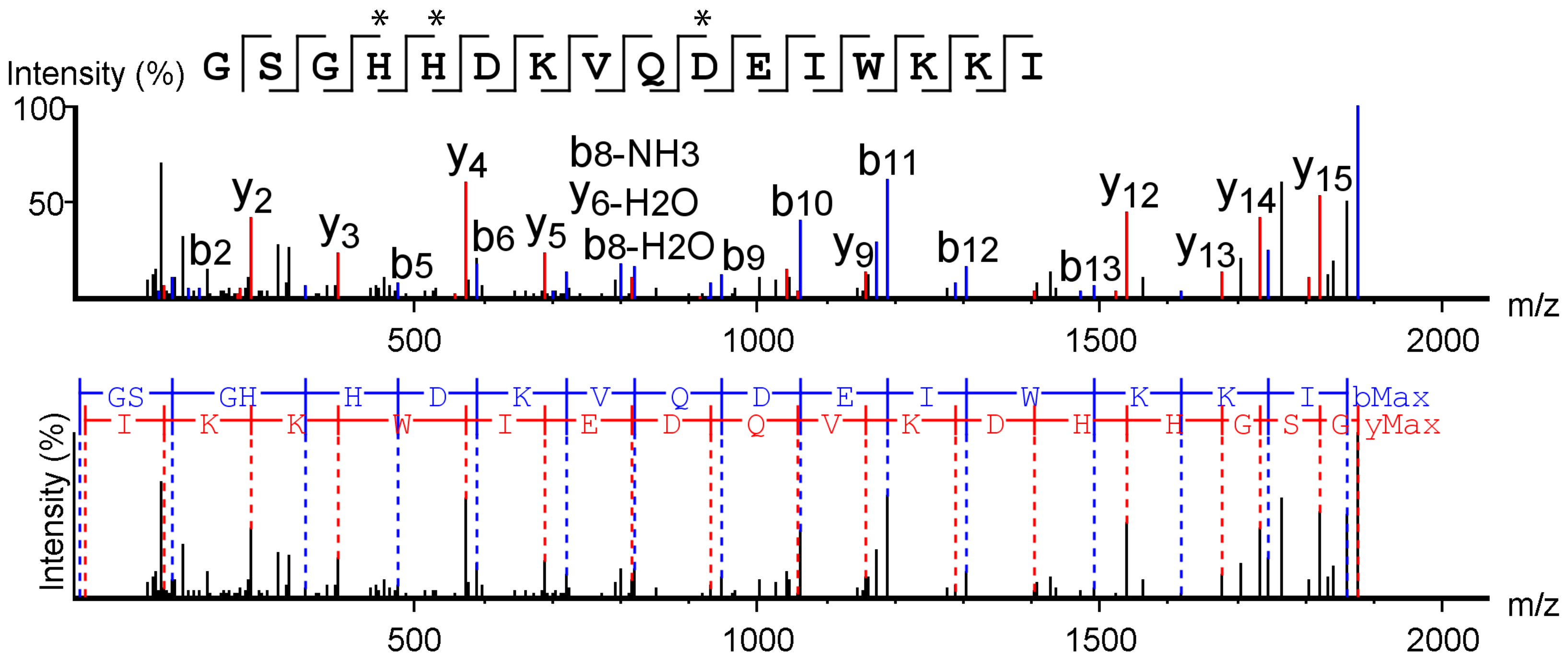

5. Hic74 [HavA]

5.1 Position (AA): **85**; Mutation **A** → **S**

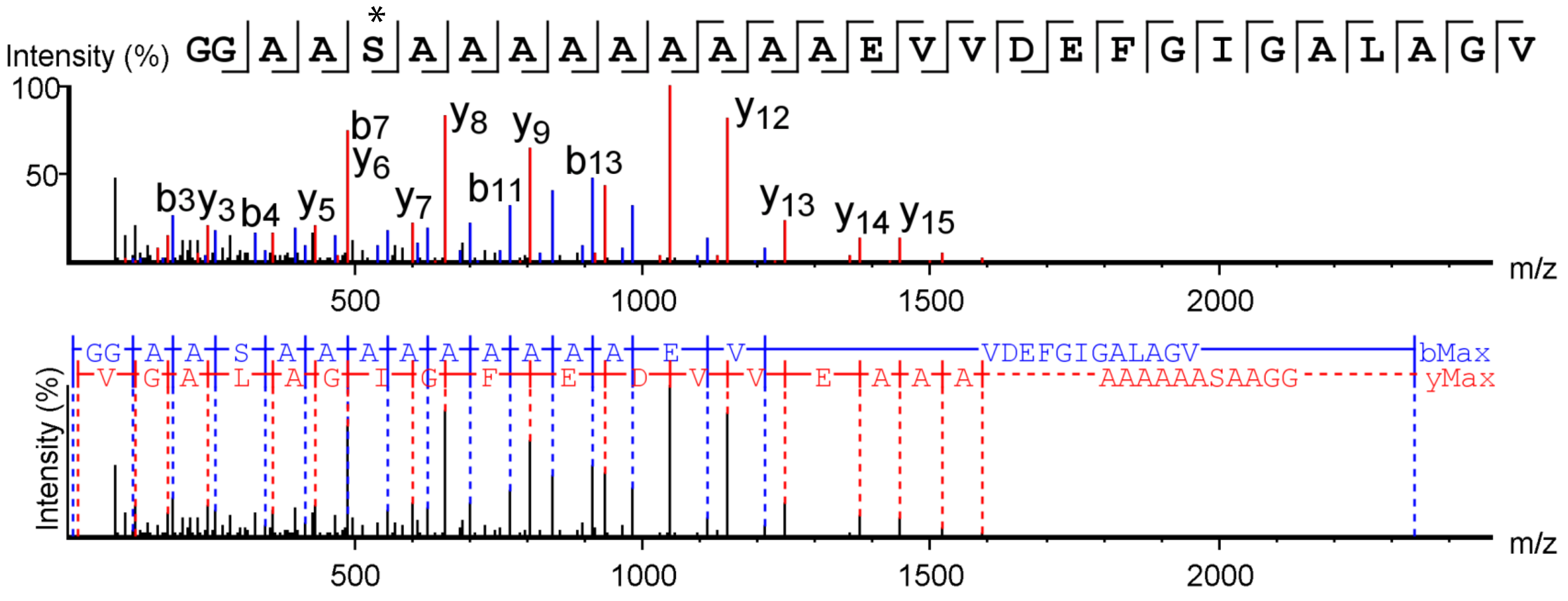

5.2 Position (AA): **85**; Mutation **A** → **S**

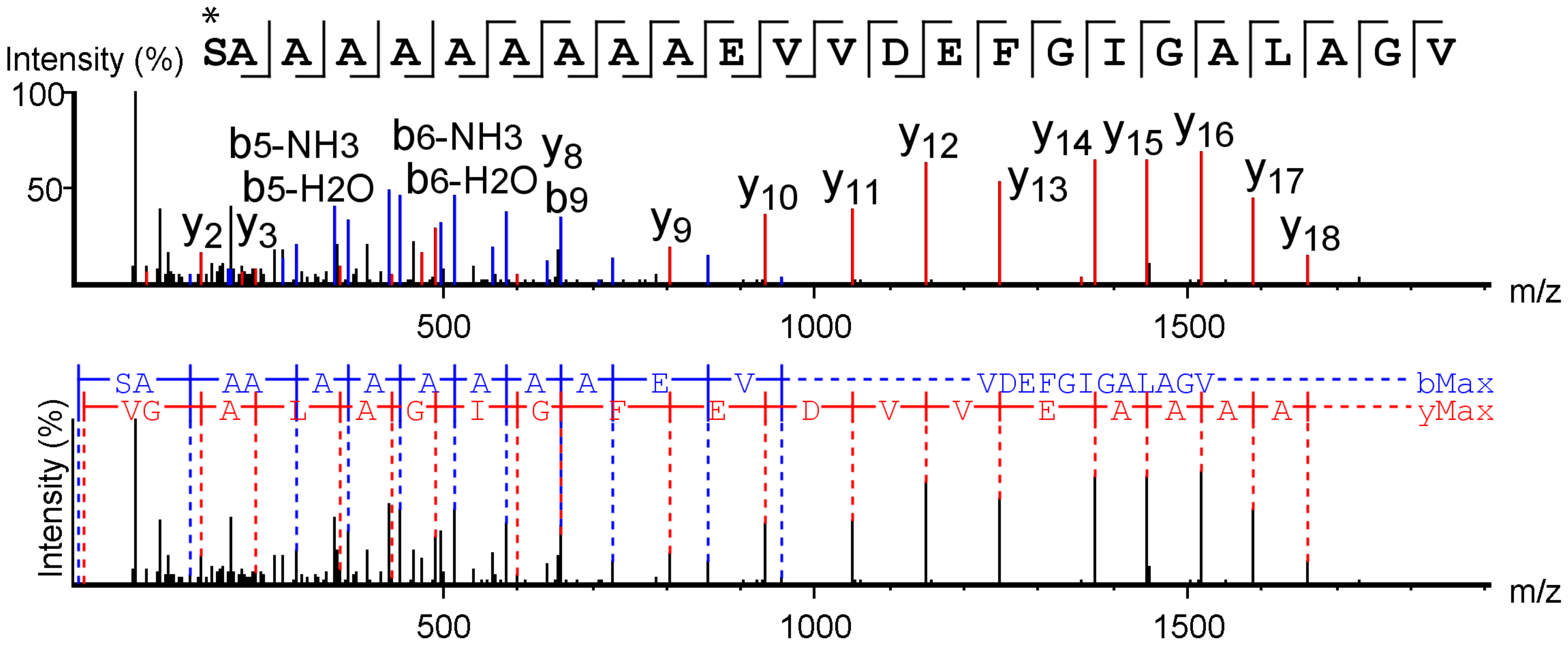

5.3 Position (AA): **111**; Mutation **G** → **A**

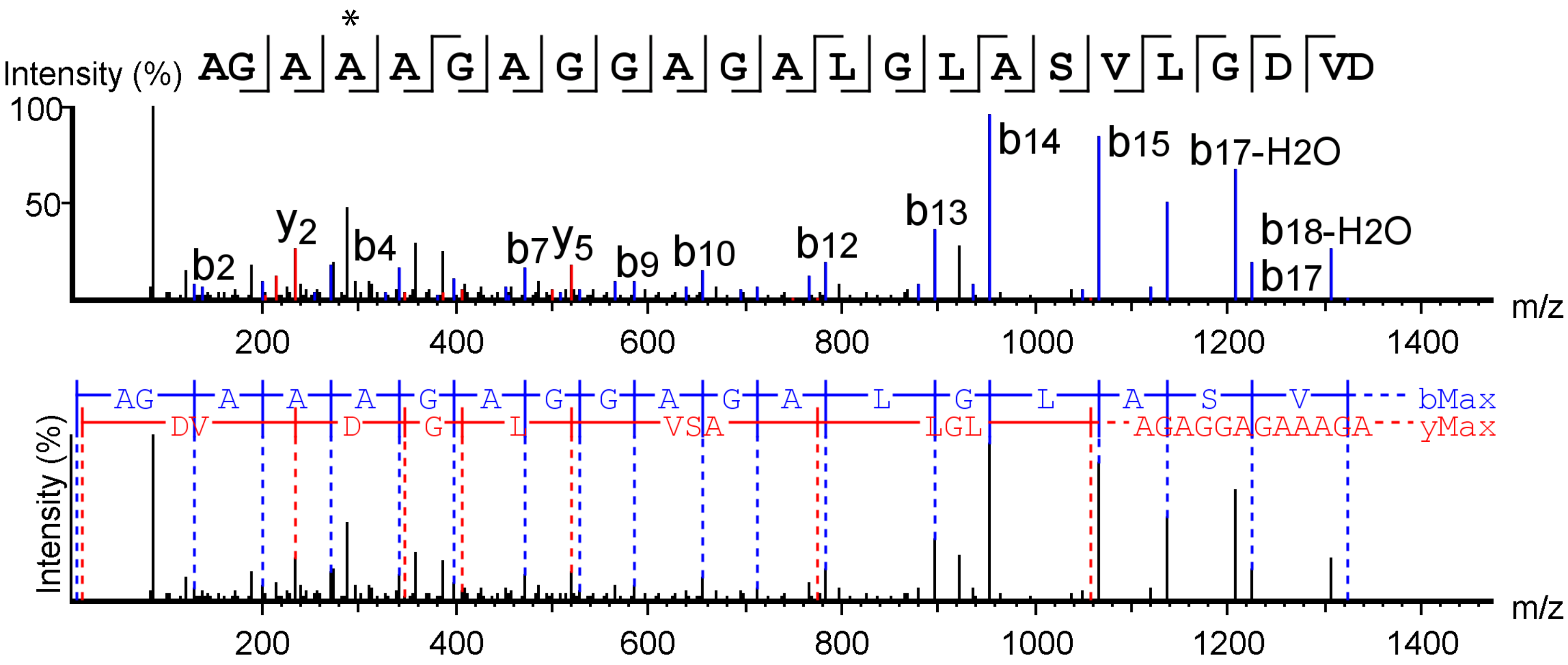

5.4 Position (AA): **111**; Mutation **G** → **A**

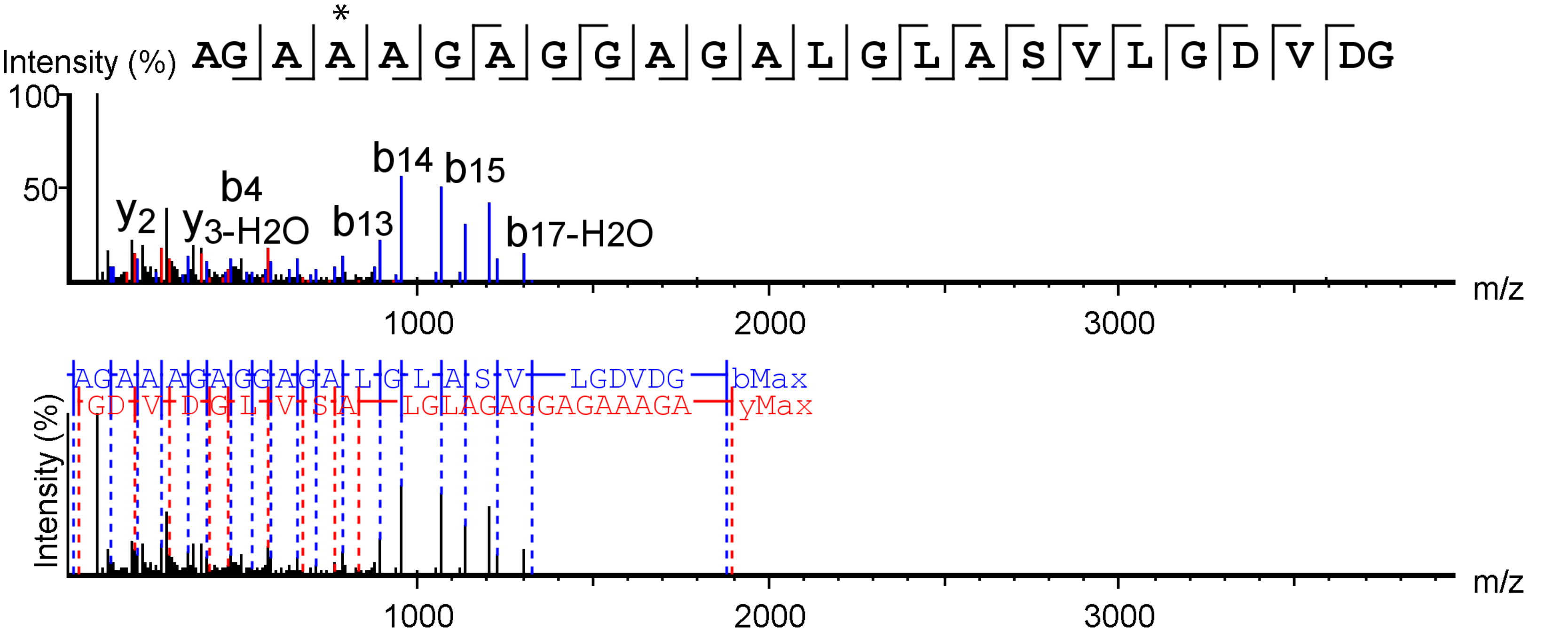

5.5 Position (AA): **172**; Mutation **E** → **Q**

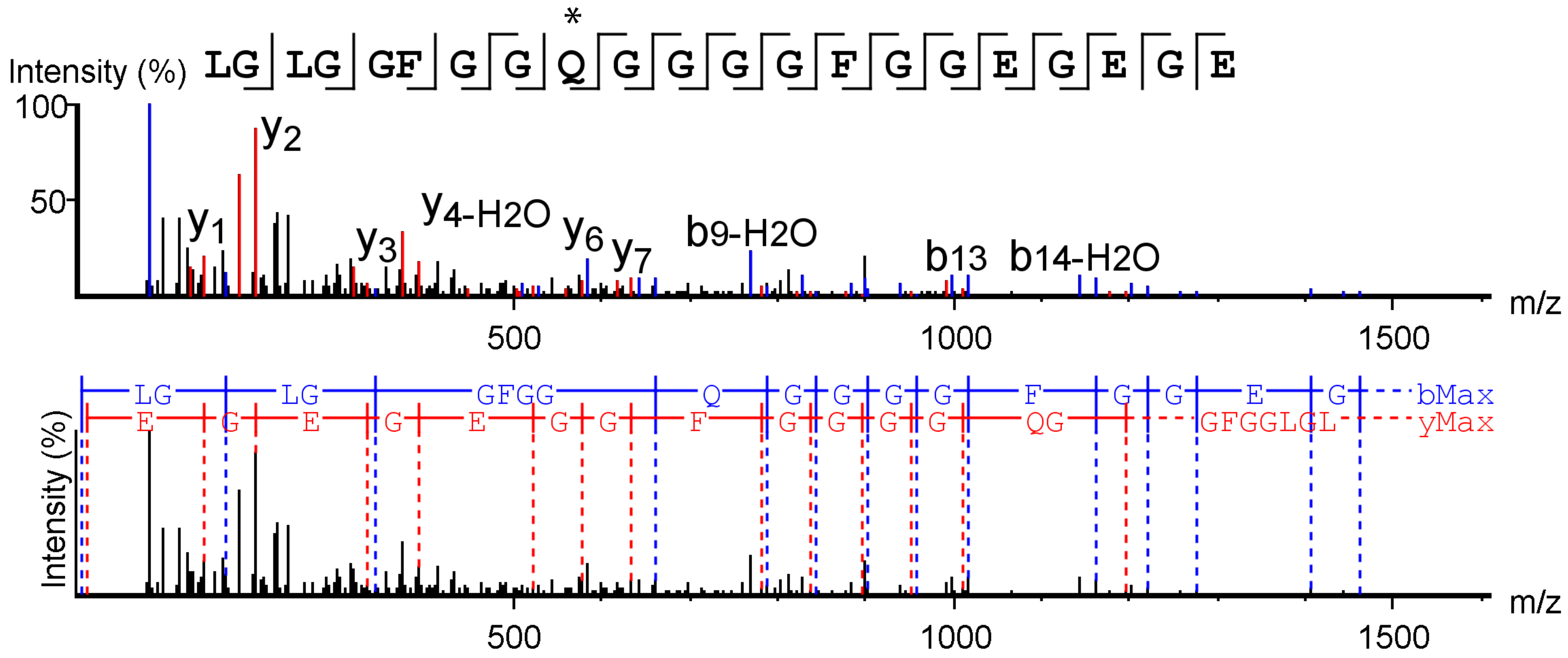

5.6 Position (AA): **289**; Mutation **V** → **L**

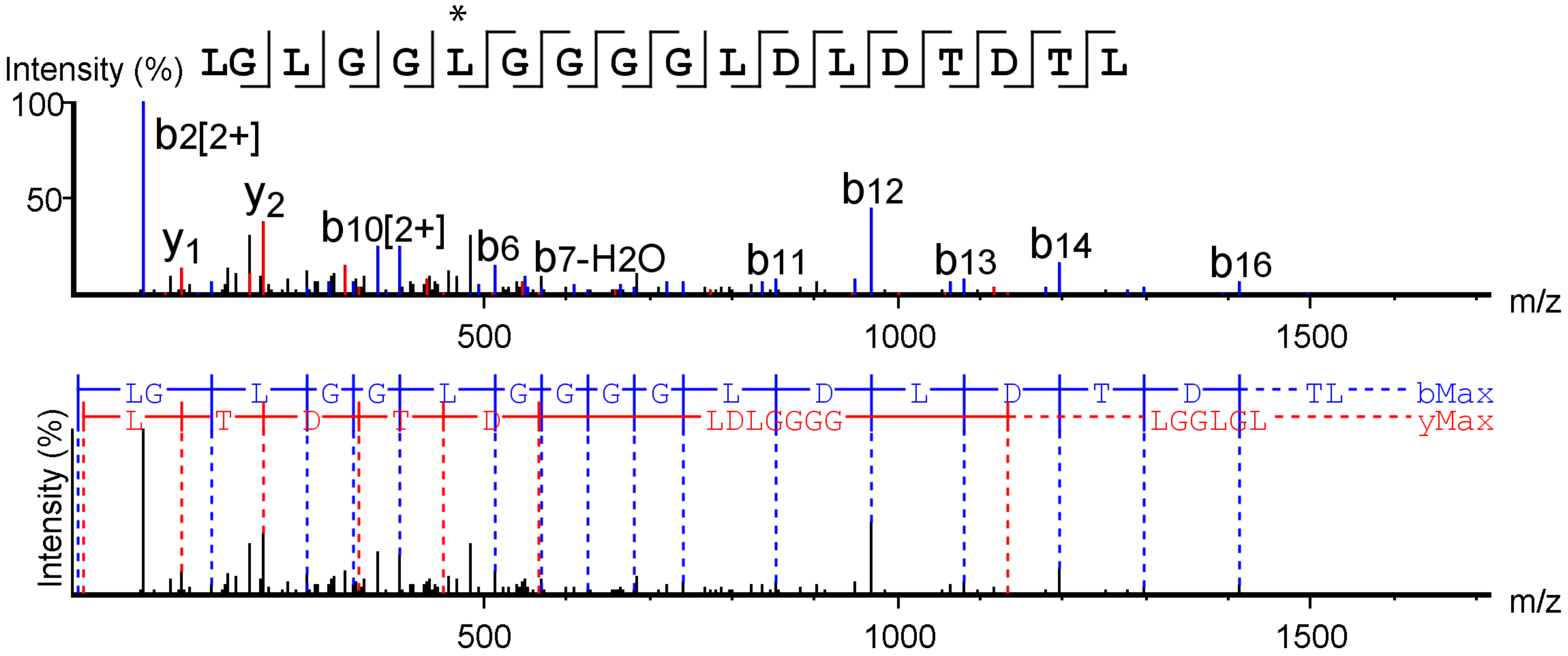

5.7 Position (AA): **289**; Mutation **V** → **L**

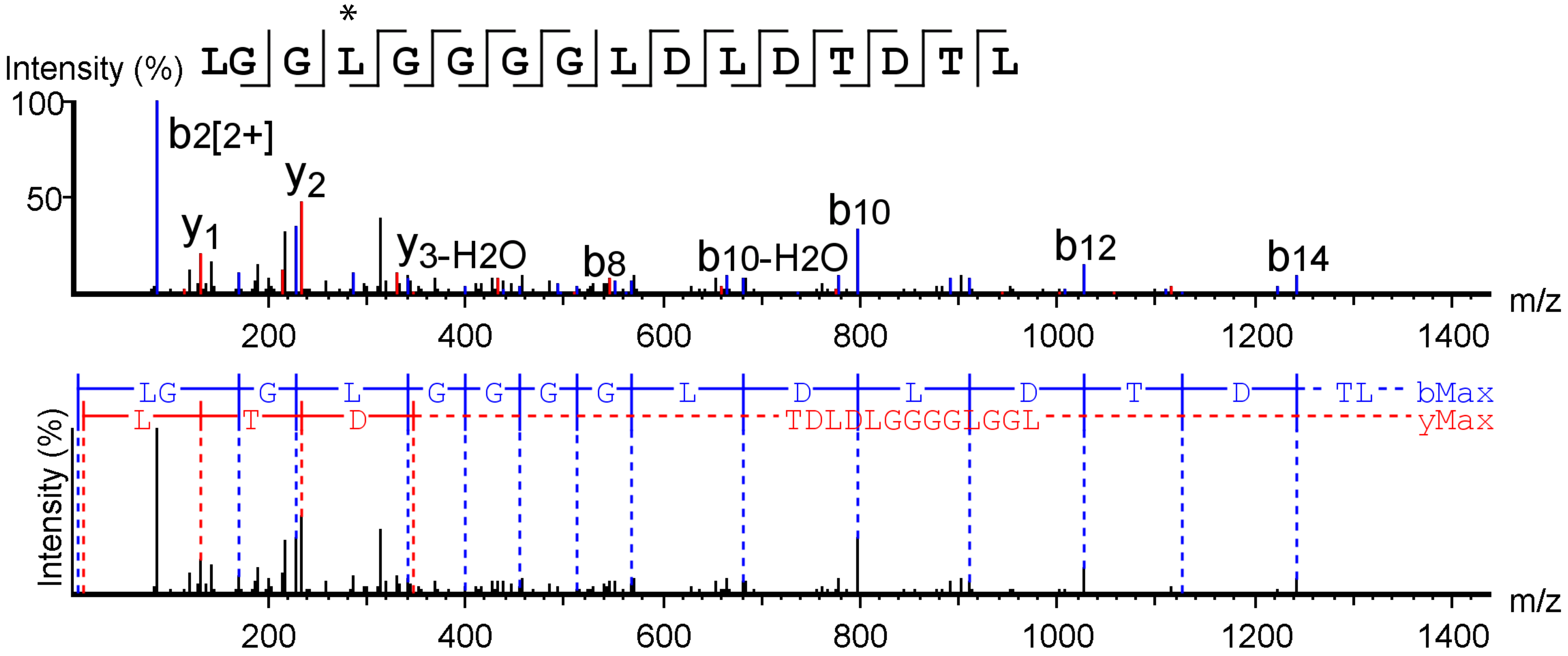

5.8 Position (AA): **310**; Mutation **I** → **F**

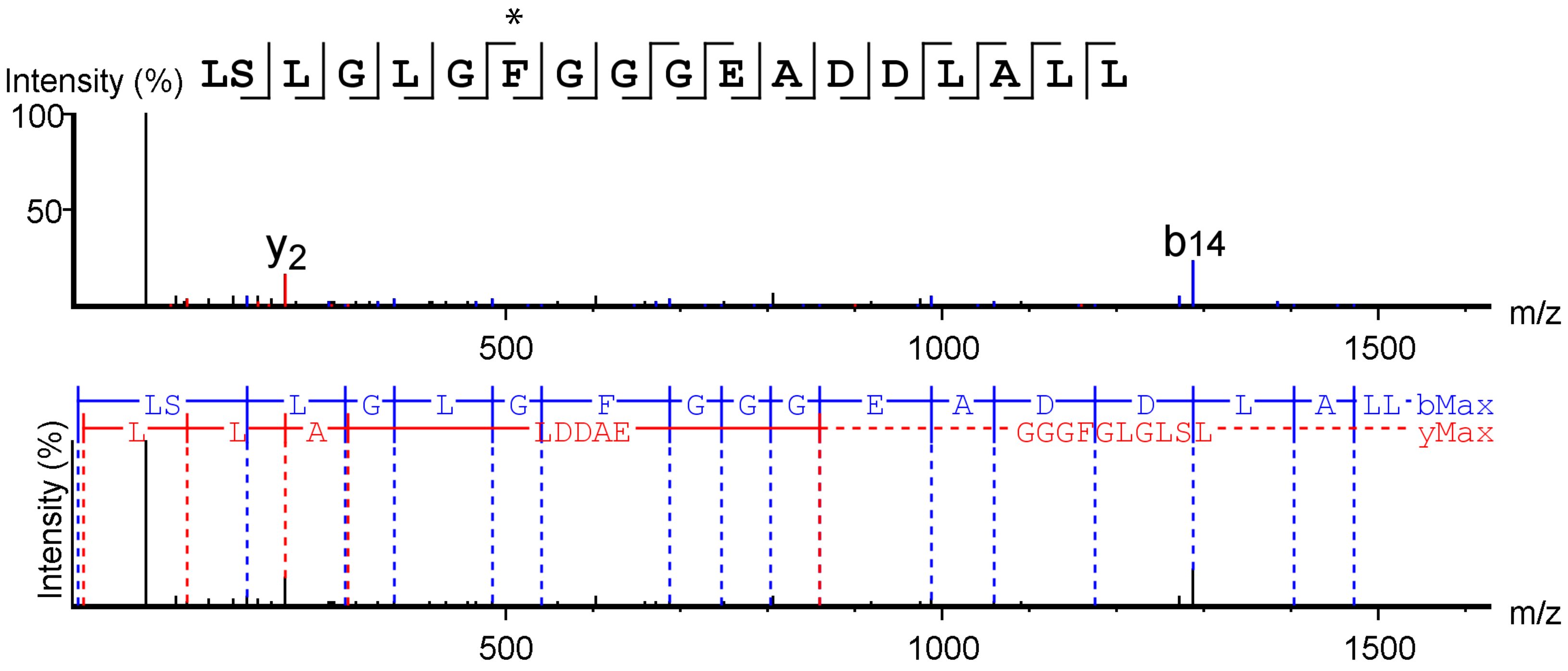

5.9 Position (AA): **822**; Mutation **R** → **G**

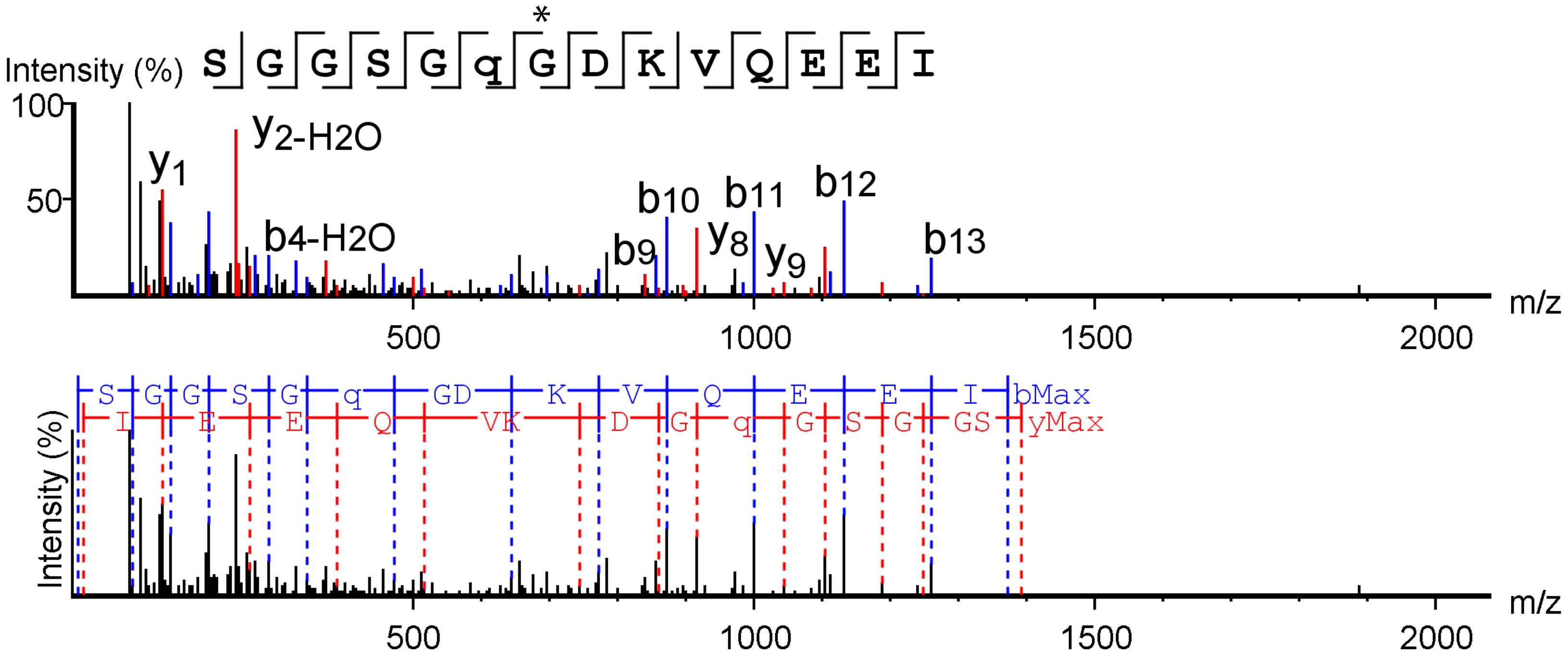

5.10 Position (AA): **822**; Mutation **R** → **G**

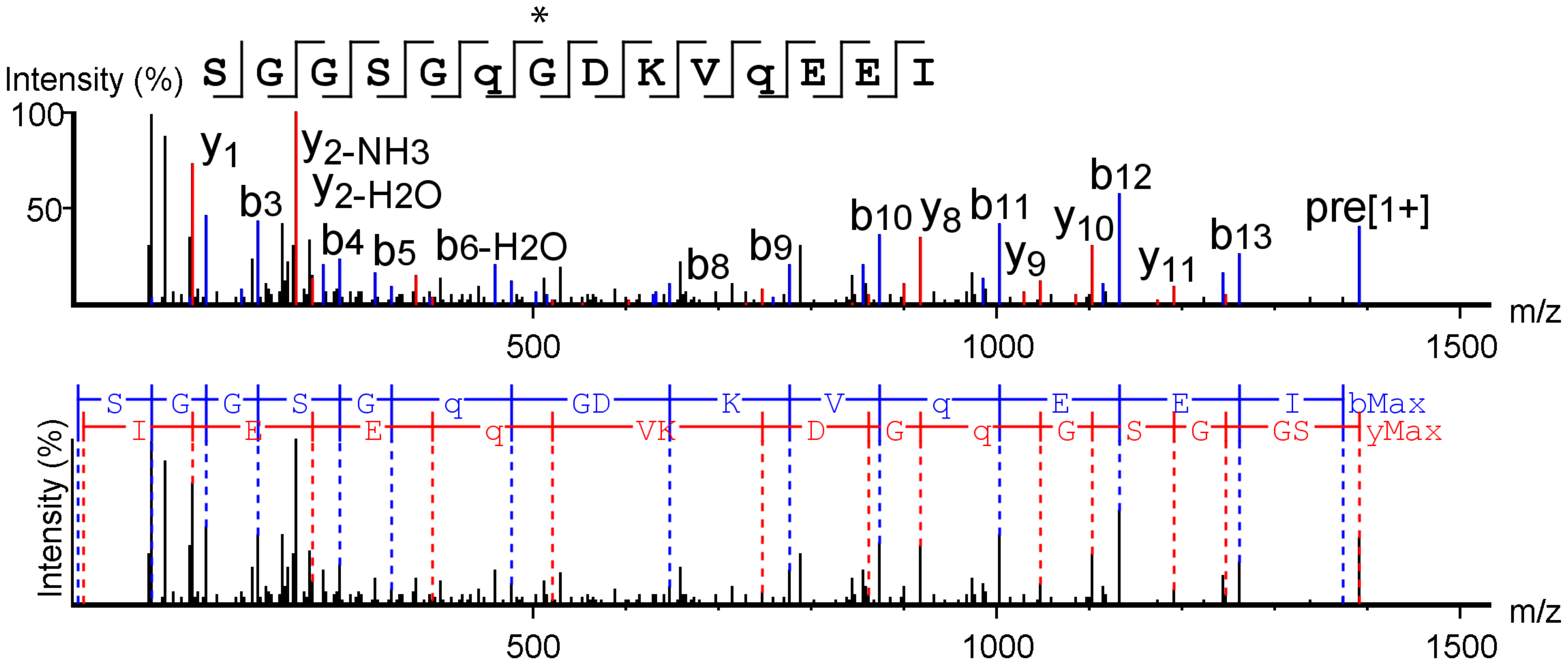

6. Hic74 [HavB]

6.1 Position (AA): **85**; Mutation **A** → **S**

108; Mutation **A** → **V**

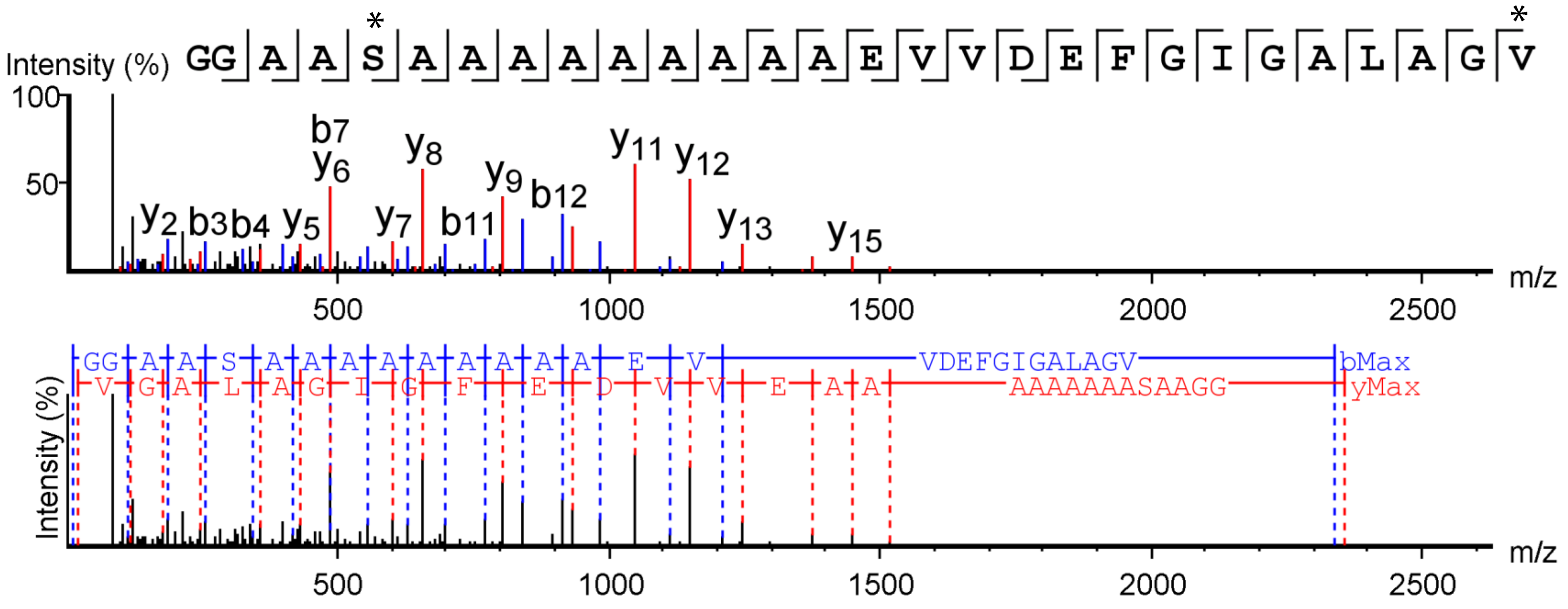

6.2 Position (AA): **289**; Mutation **V** → **L**

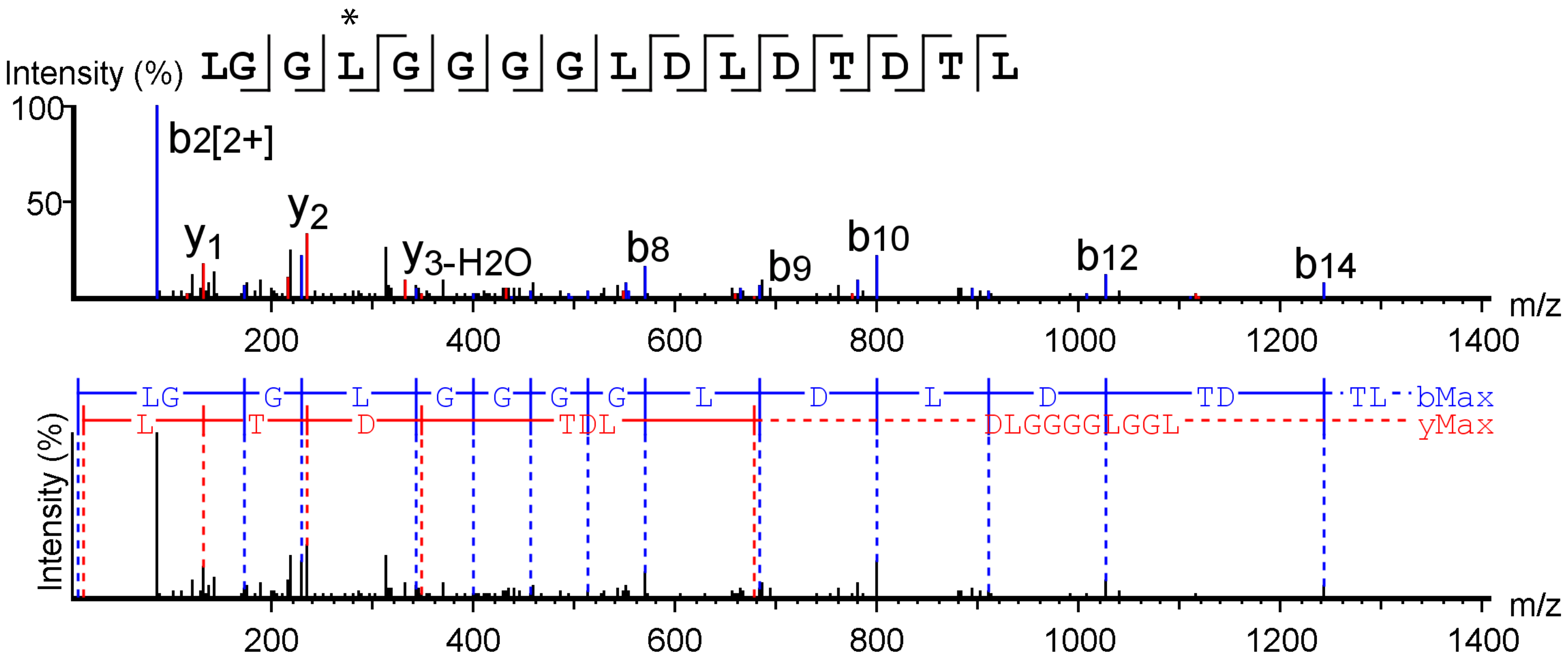

6.3 Position (AA): **310**; Mutation **I** → **F**

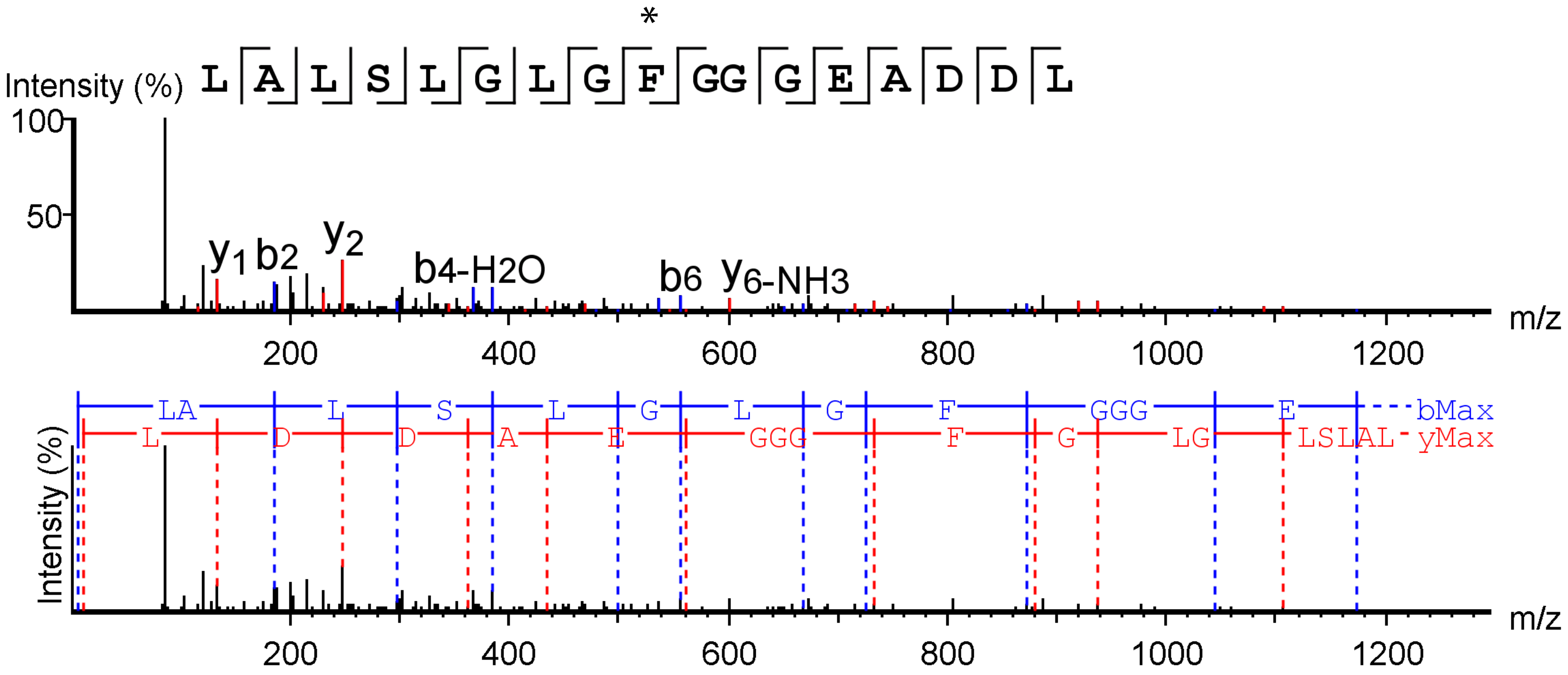

6.4 Position (AA): **822**; Mutation **R** → **G**

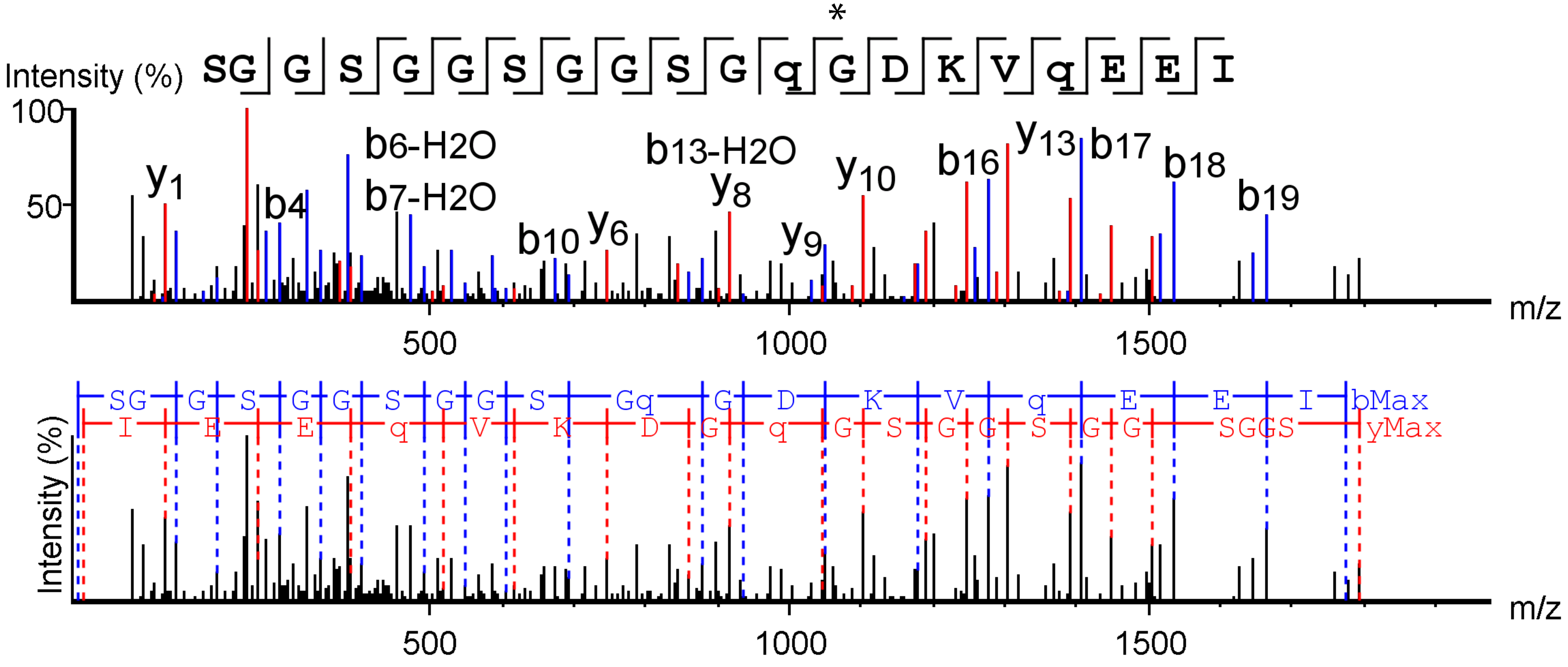

6.5 Position (AA): **822**; Mutation **R** → **G**

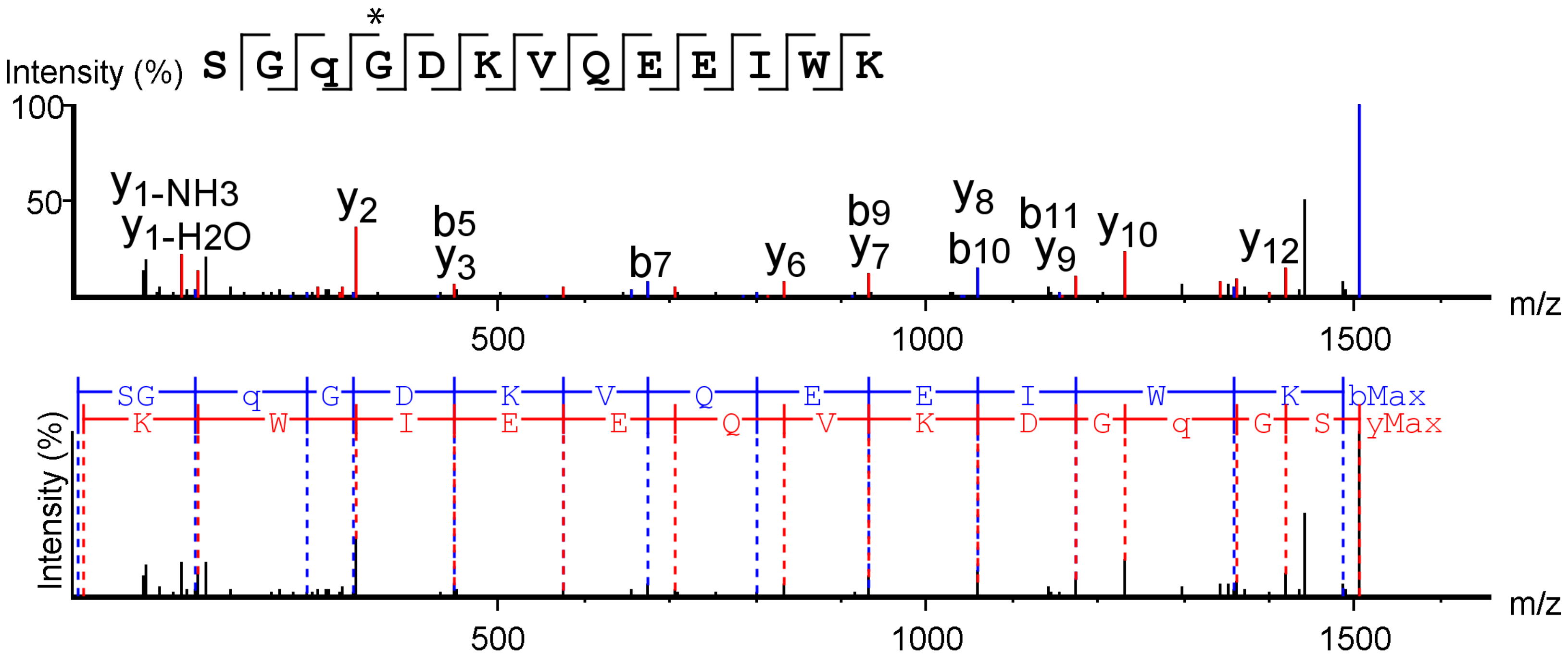

7. Hic74 [HavC]

7.1 Position (AA): **85**; Mutation **A** → **S**
108; Mutation **A** → **V**

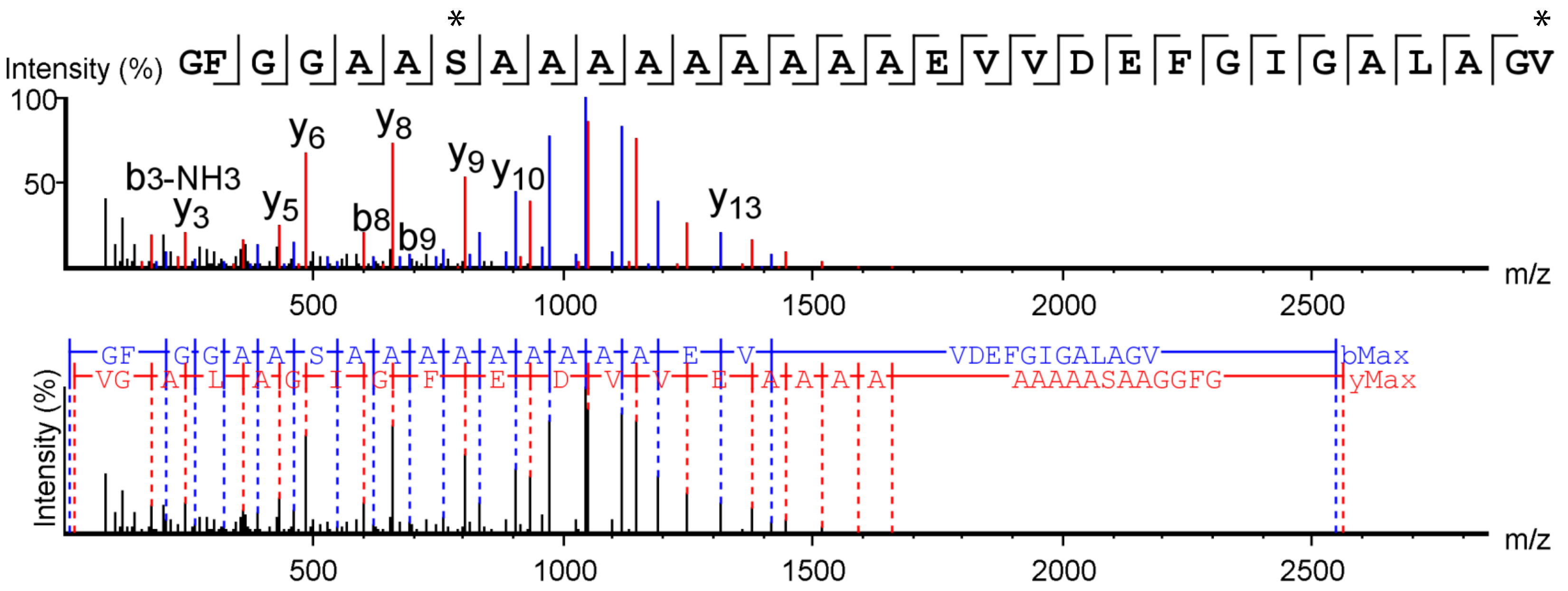

7.2 Position (AA): **85**; Mutation **A** → **S**

108; Mutation **A** → **V**

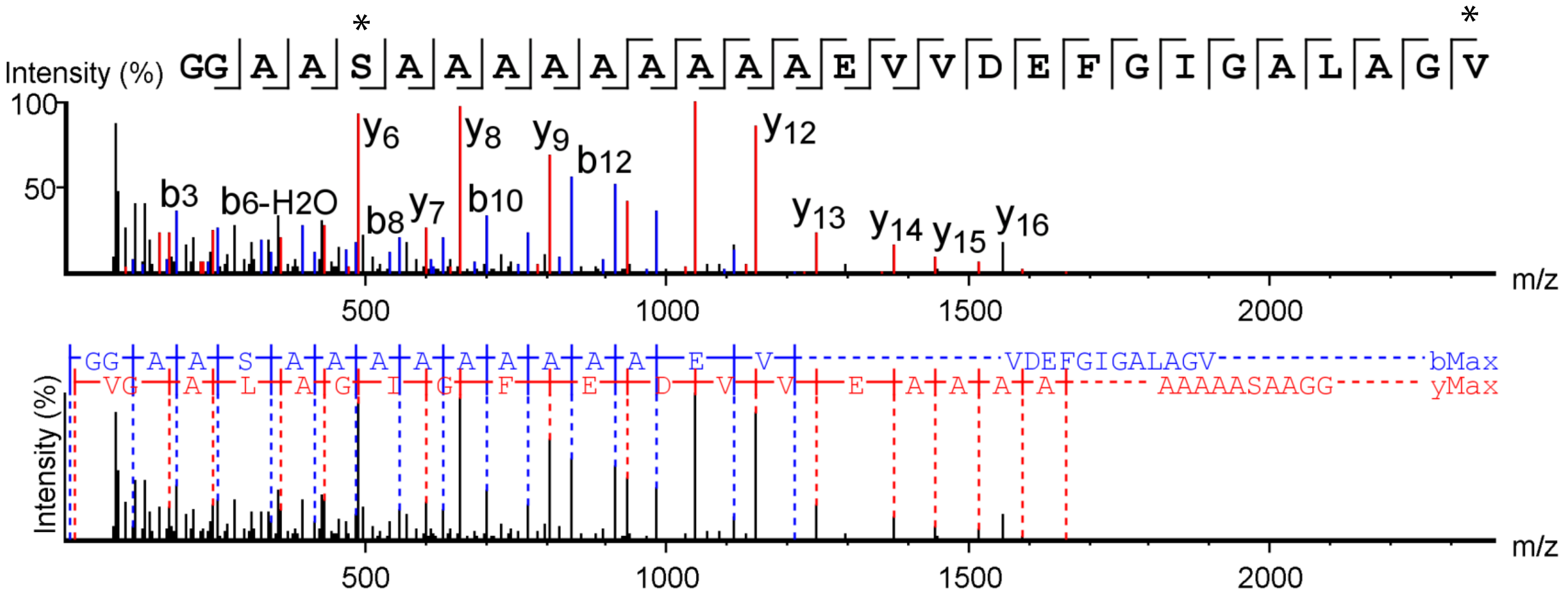

7.3 Position (AA): **111**; Mutation **G** → **A**

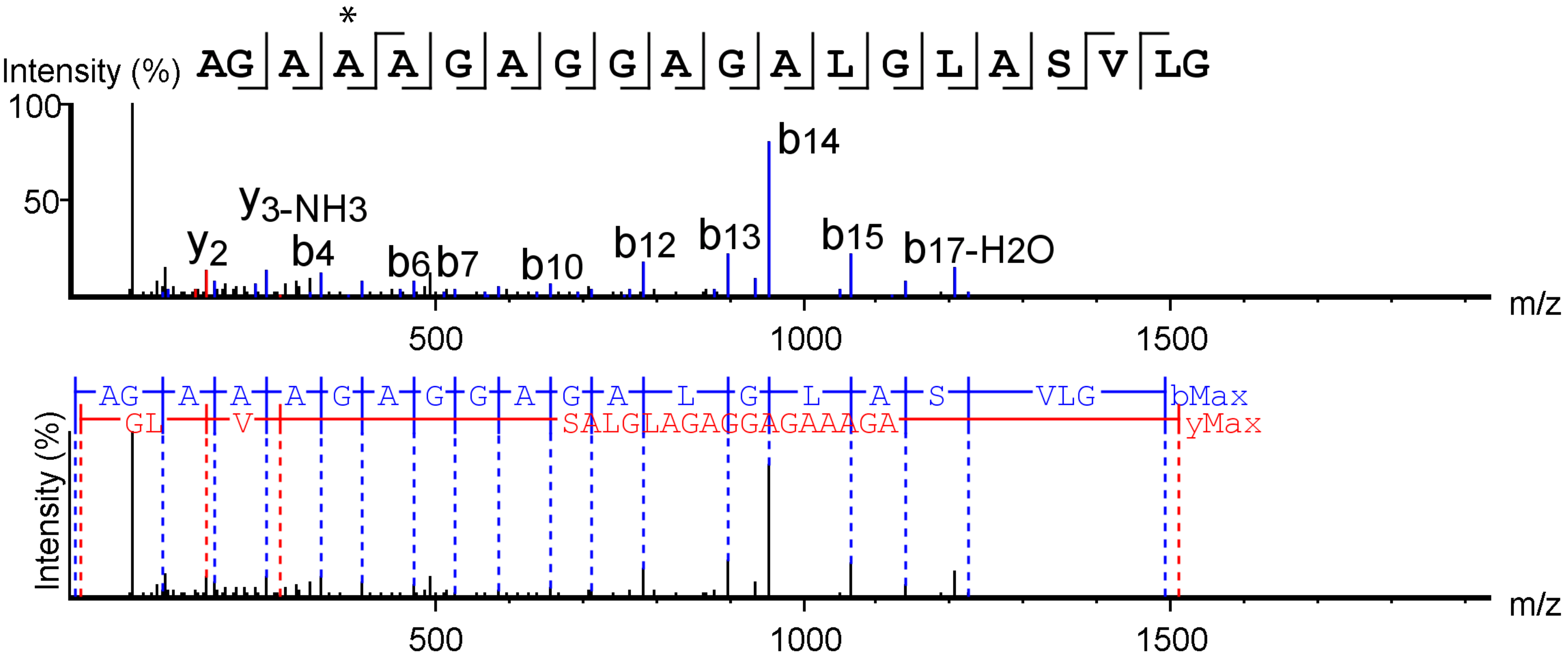

7.4 Position (AA): **111**; Mutation **G** → **A**

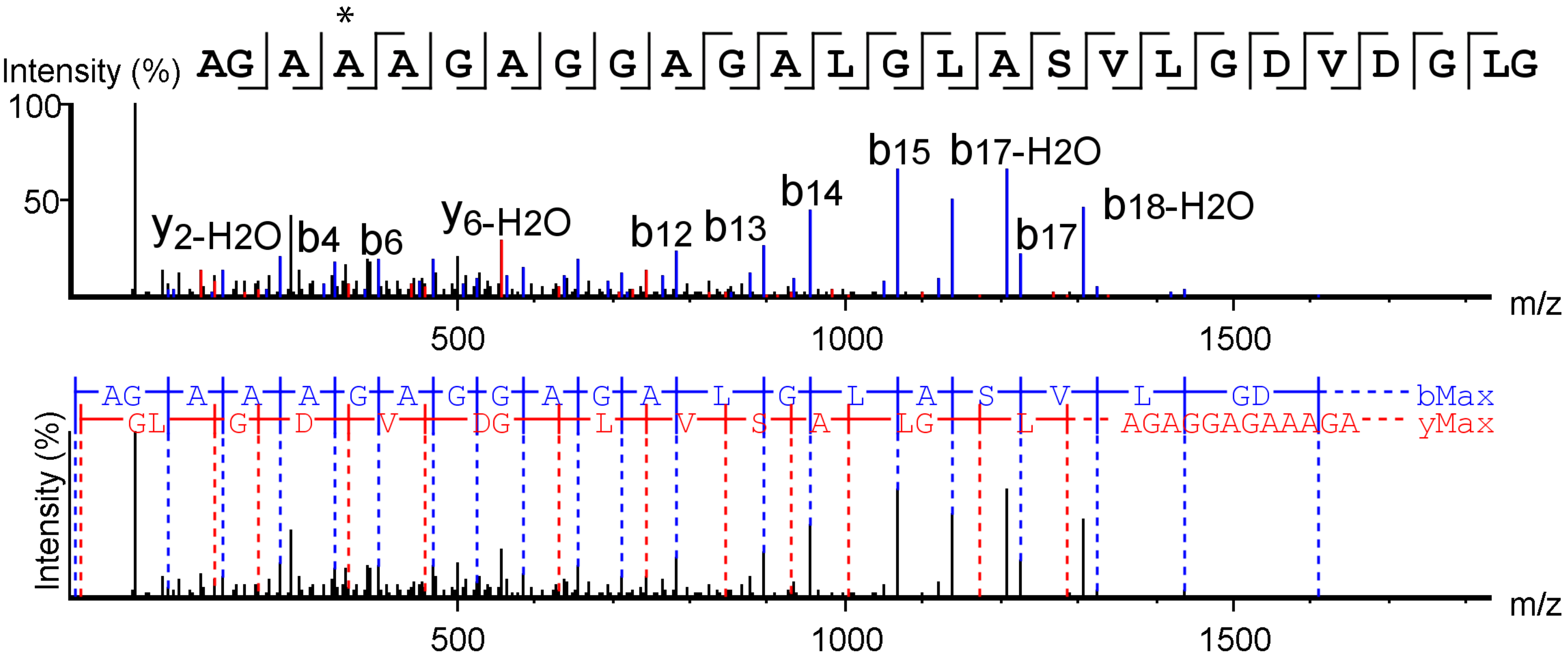

7.4 Position (AA): **163**; Mutation **S** → **G**

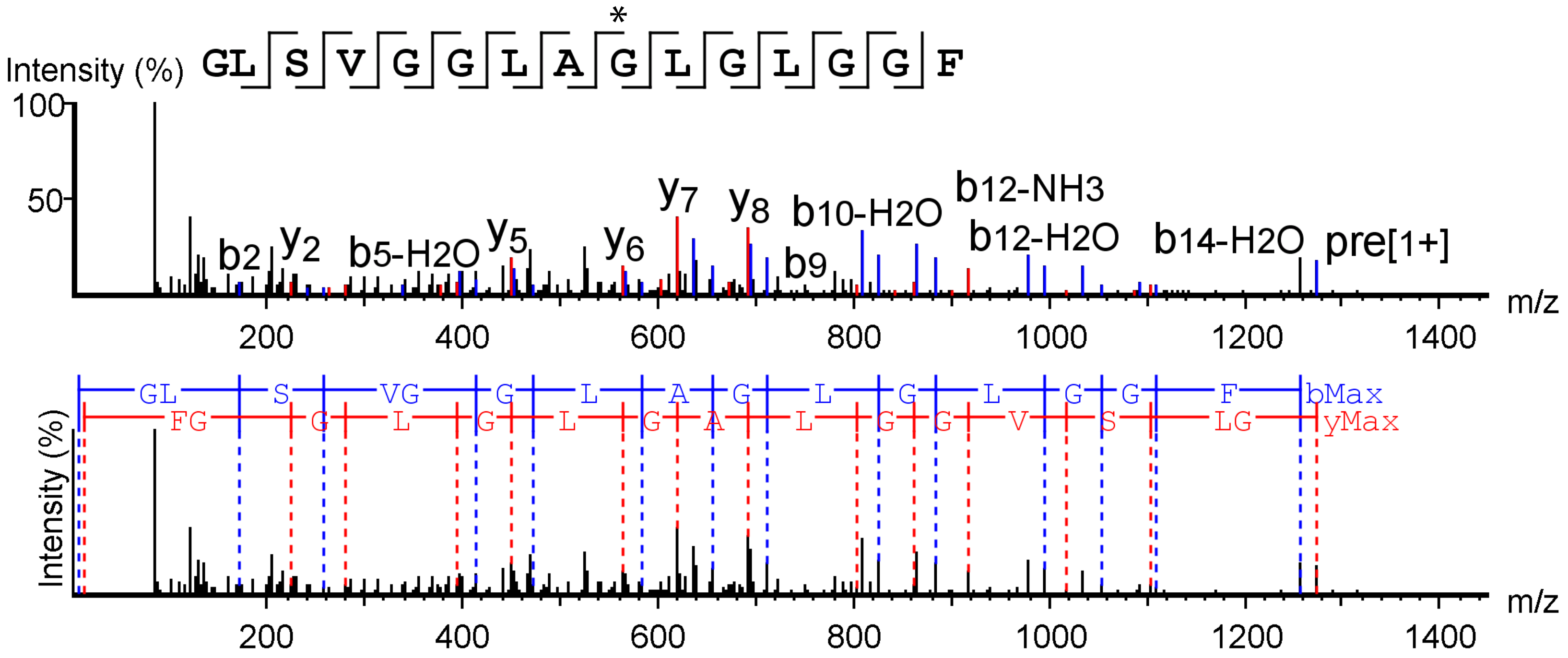

7.5 Position (AA): **163**; Mutation **S** → **G**

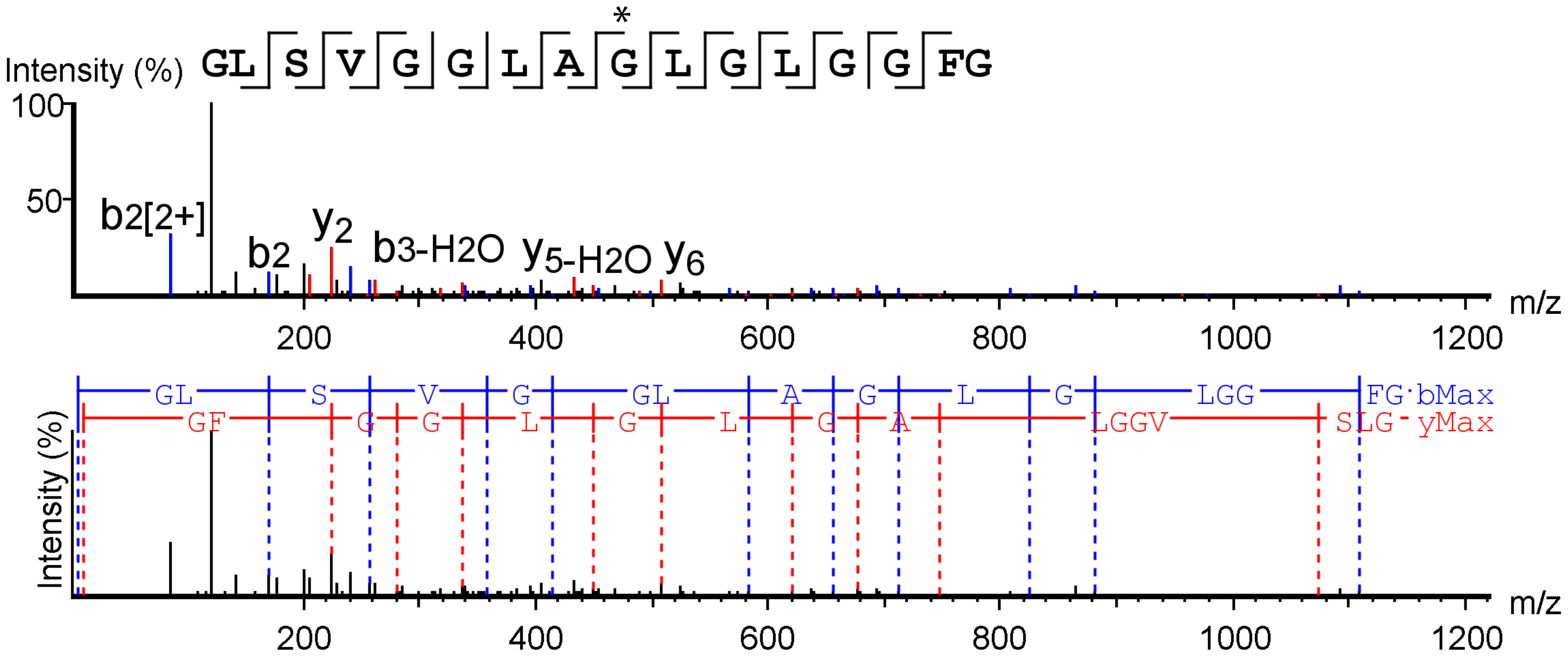

7.6 Position (AA): **289**; Mutation **V** → **L**

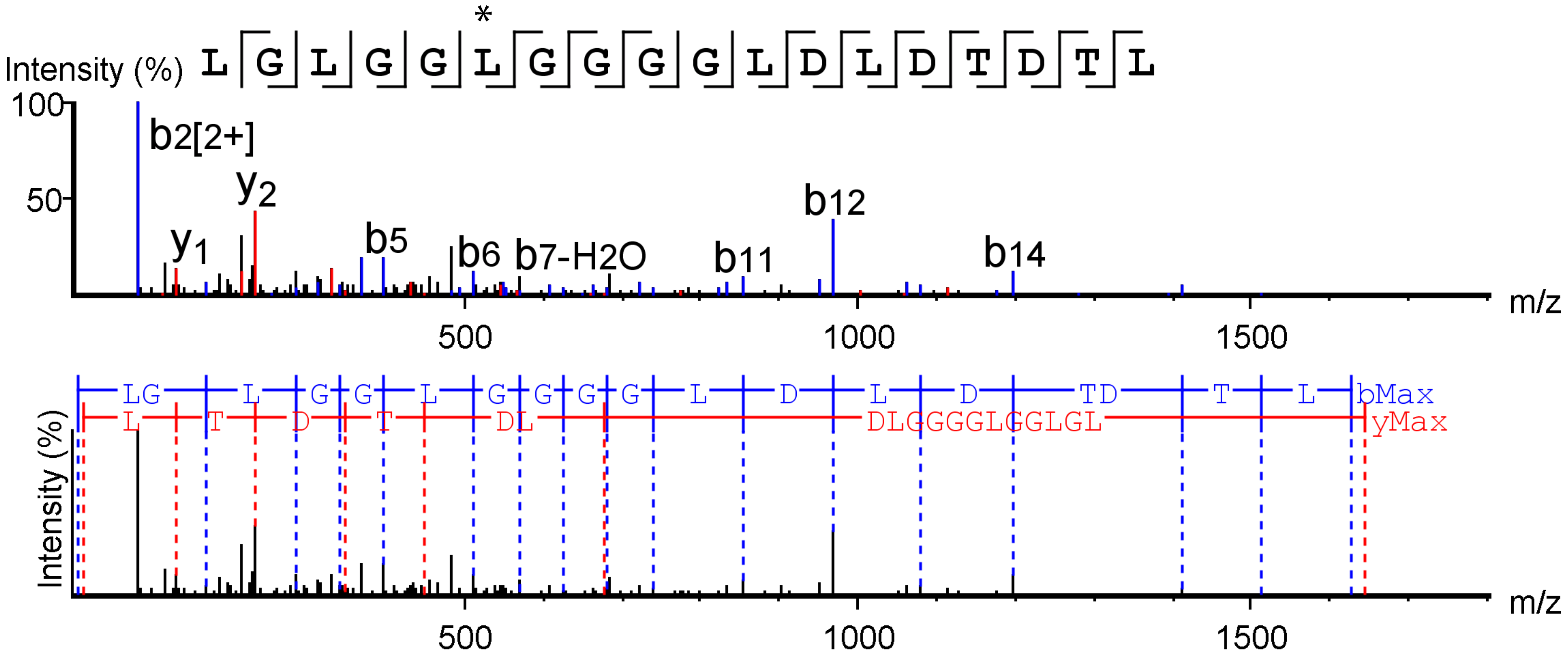

7.7 Position (AA): **289**; Mutation **V** → **L**

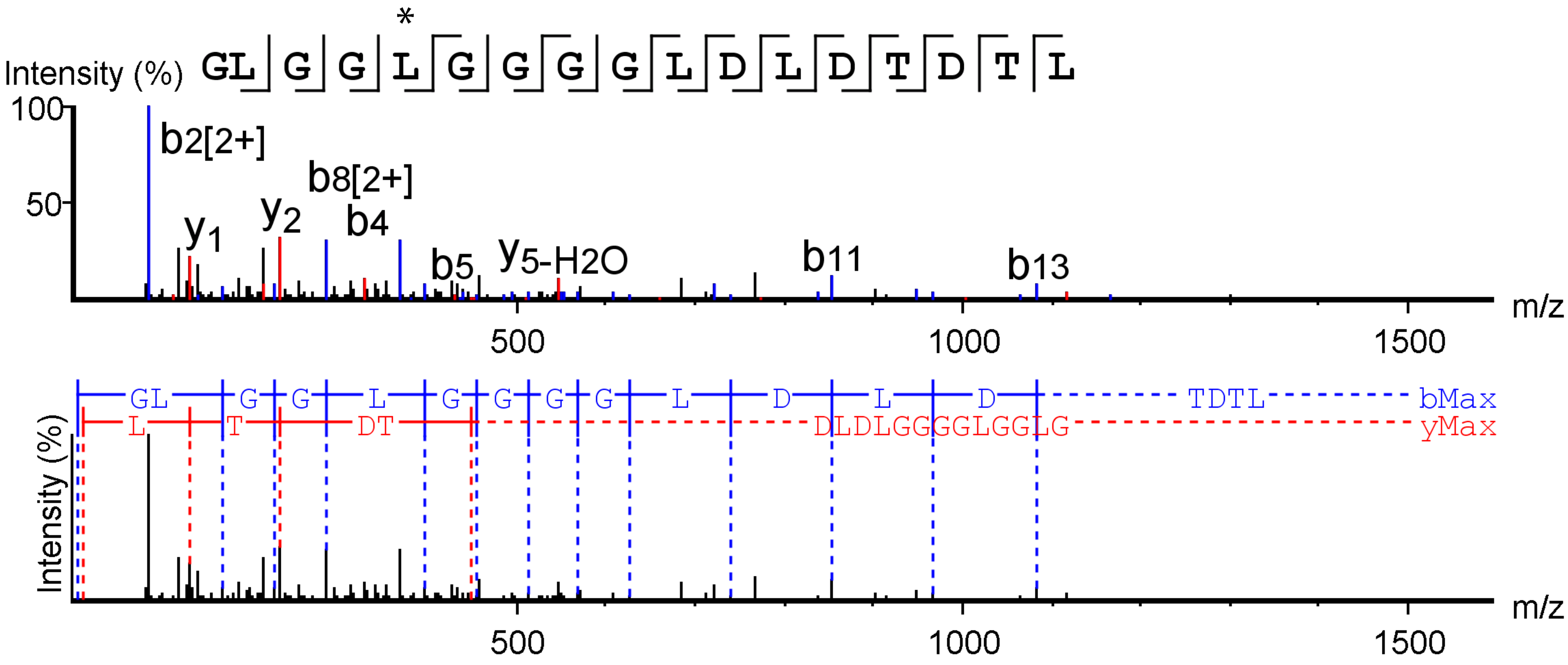

7.8 Position (AA): **310**; Mutation **I** → **F**

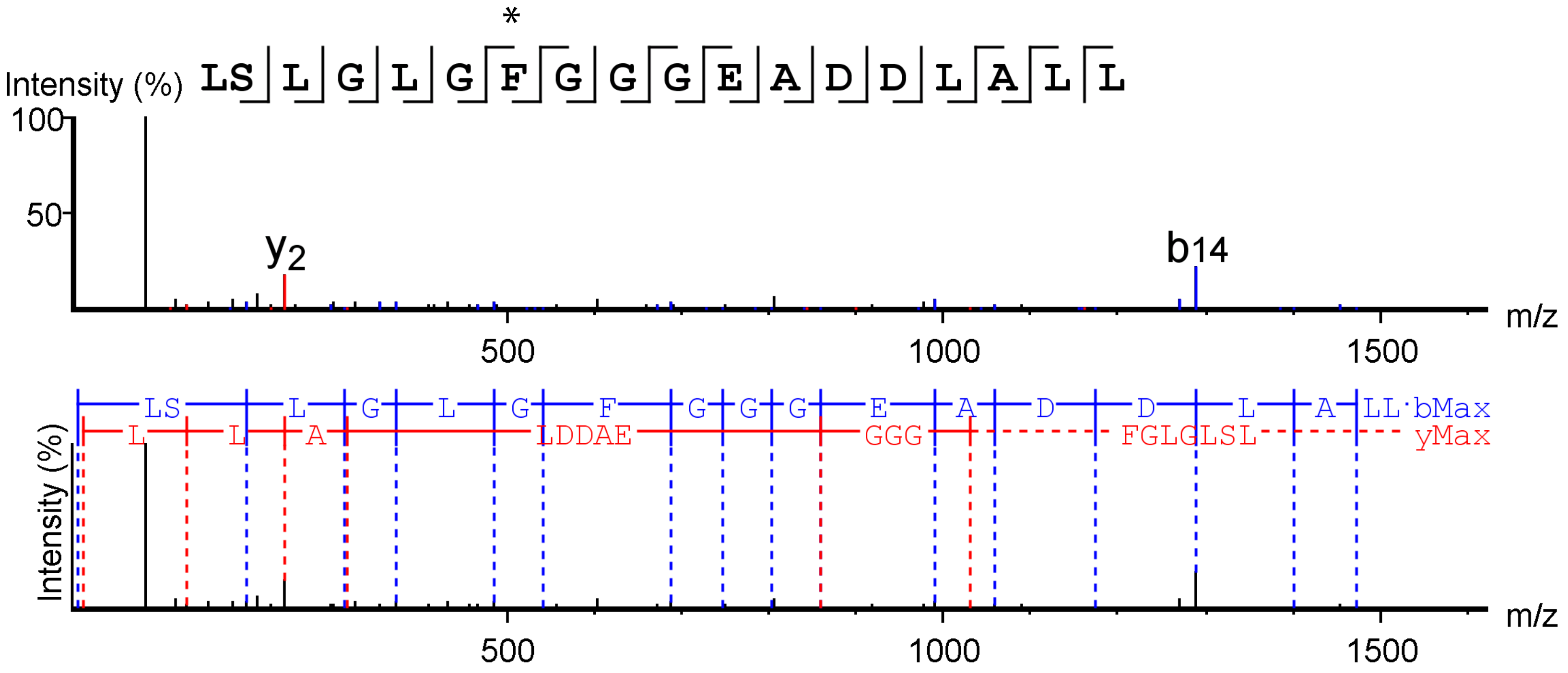

7.9 Position (AA): **310**; Mutation **I** → **F**

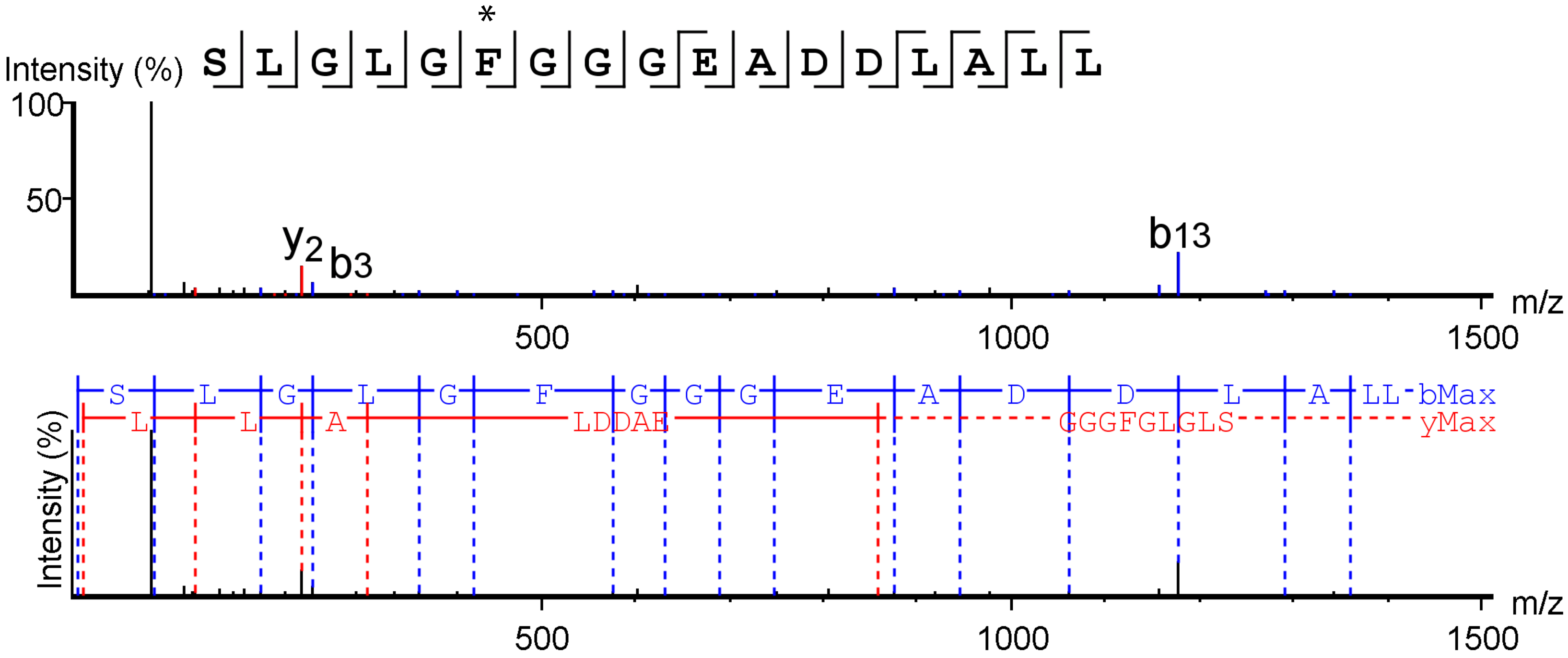

7.10 Position (AA): **804**; Mutation **A** → **S**

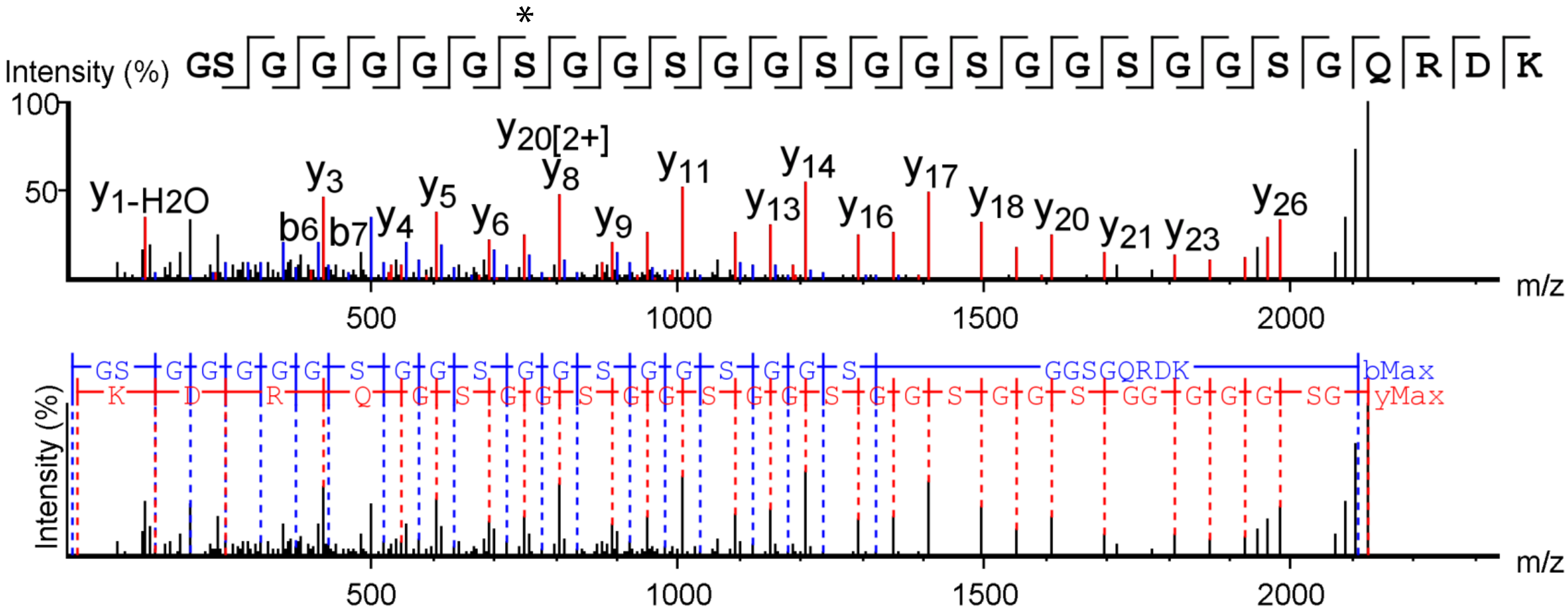

7.11 Position (AA): **804**; Mutation **A** → **S**

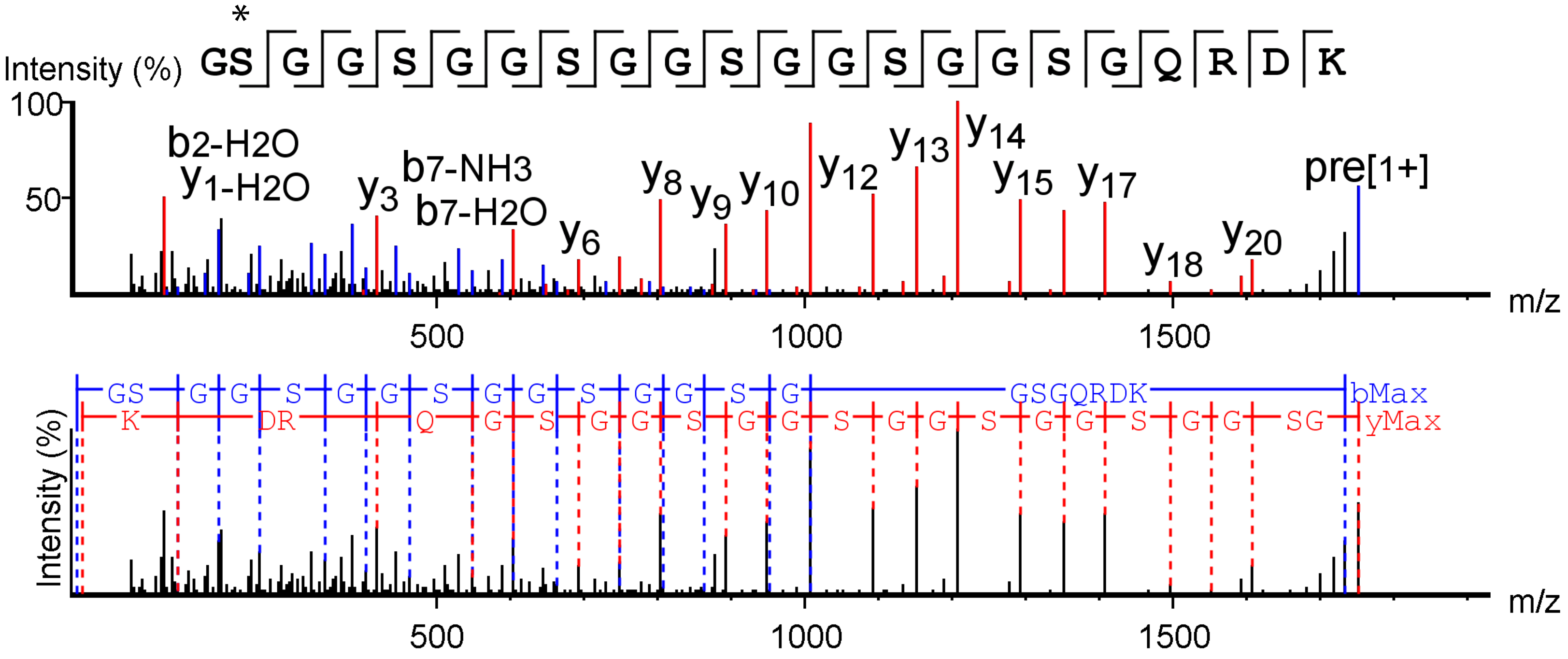

7.12 Position (AA): **822**; Mutation **R** → **G**

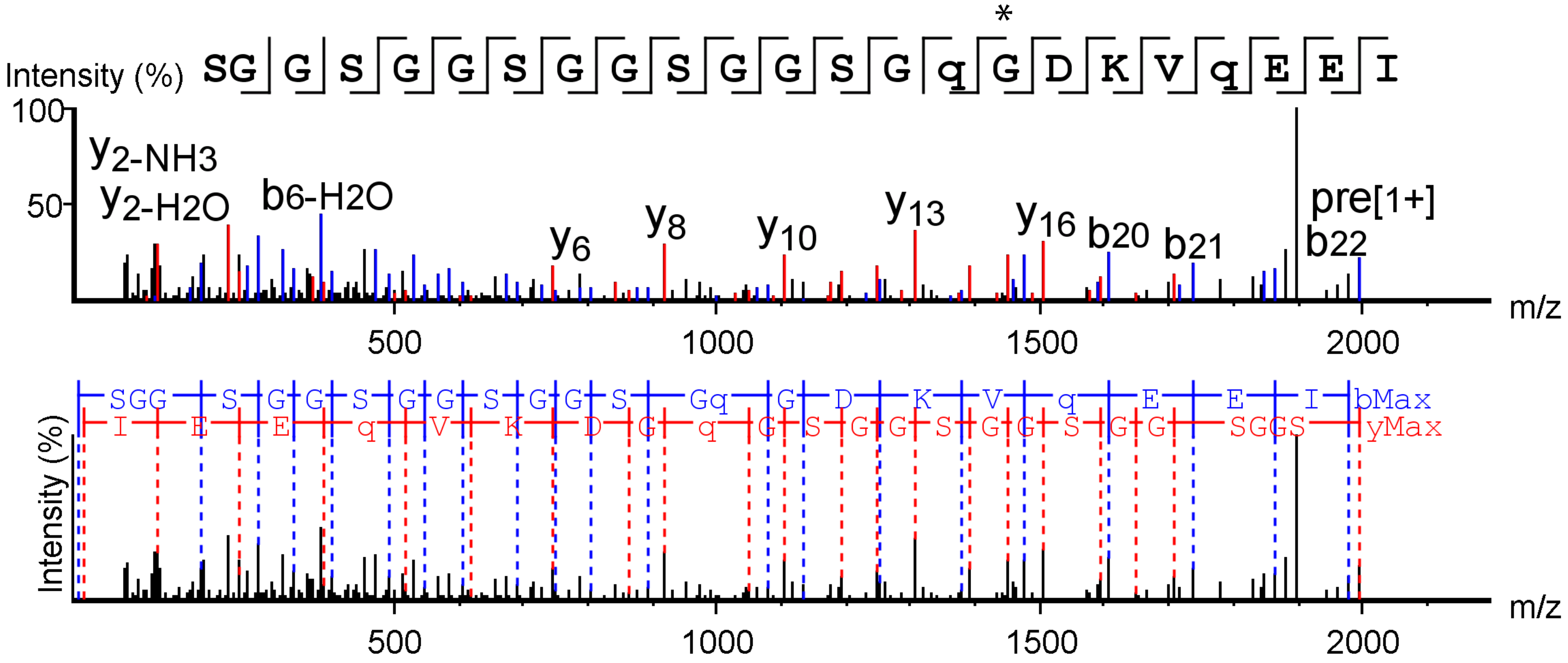

7.13 Position (AA): **822**; Mutation **R** → **G**

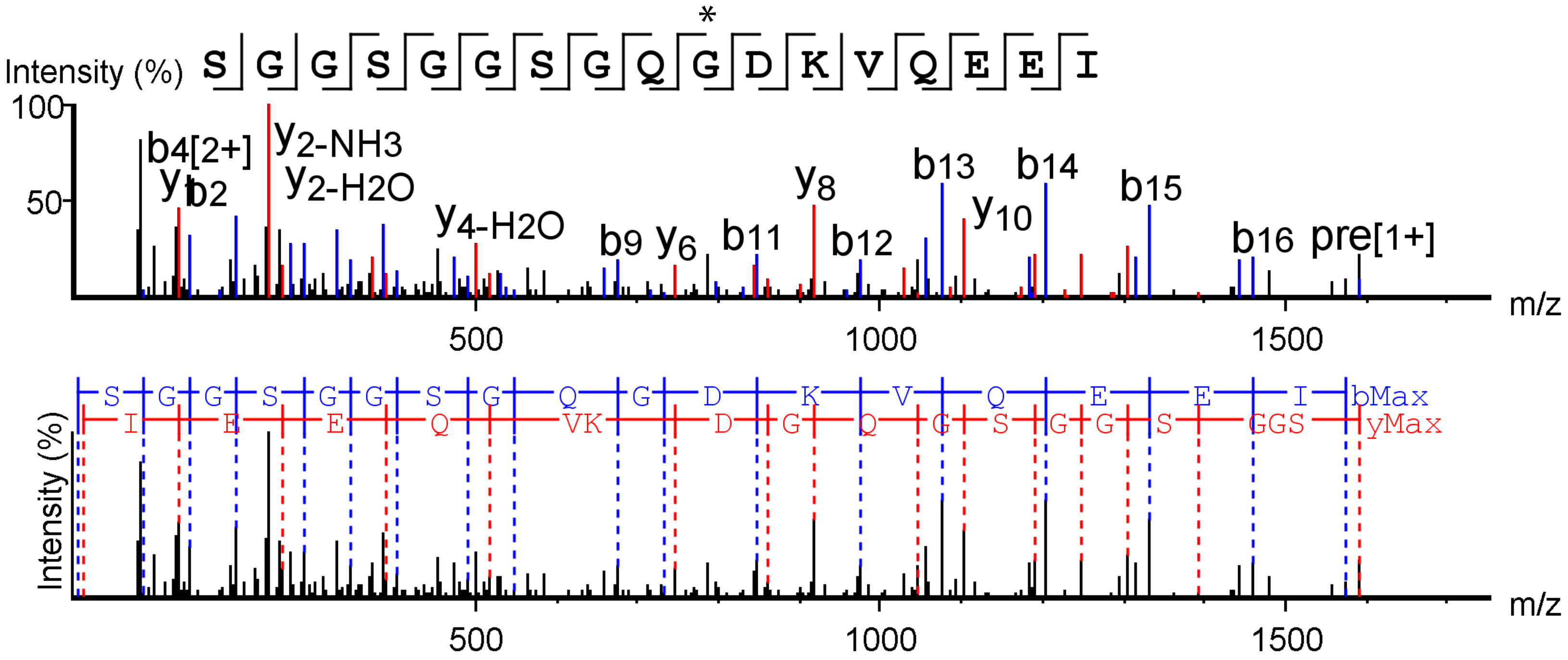

Supplement: Figure 6—source data 1. [file elife-45644-fig6-data1.pdf]
